# TrkC protects against osteoarthritis progression by maintaining articular cartilage homeostasis

**DOI:** 10.7150/ijbs.108832

**Published:** 2025-05-27

**Authors:** Yongyun Chang, Keyu Kong, Hua Qiao, Minghao Jin, Xinru Wu, Wenxuan Fan, Jingwei Zhang, Yansong Qi, Yongsheng Xu, An Qin, Zanjing Zhai, Huiwu Li

**Affiliations:** 1 Shanghai Key Laboratory of Orthopaedic Implants, Department of Orthopaedics, Ninth People's Hospital, Shanghai Jiao Tong University School of Medicine, Shanghai, China; 2 Department of Orthopedics, Inner Mongolia People's Hospital, Hohhot, China

**Keywords:** Osteoarthritis, TrkC, NT3, Cartilage degeneration, PI3K/Akt signaling pathway.

## Abstract

Osteoarthritis (OA) is a degenerative disease with a series of metabolic changes accompanied by chondrocyte apoptosis. Chondrocytes express multiple receptors for neurotrophin, however, the role of neurotrophin receptor in chondrocyte metabolism remains unelucidated. Here, we first clarify the role of neurotrophin 3 (NT3) and its receptor tropomyosin receptor kinase C (TrkC) of chondrocytes in OA pathogenesis, using inducible TrkC-deficient mice (TrkC^fl/fl^; Col2a1-CreER^T2^ mice). Our findings show that TrkC levels are decreased in the chondrocytes and cartilage of patients with OA and OA-model mice. Chondrocyte-specific TrkC deficiency aggravates cartilage destruction during OA development. However, intra-articular TrkC-overexpressing adeno-associated virus (AAV) injection delays experimental OA progression. TrkC deficiency leads to decreased anabolic and increased catabolic activities in chondrocytes and stimulates chondrocyte apoptosis, thereby accelerating OA progression. Whereas TrkC overexpression rescues the imbalance between extracellular matrix synthesis and degradation and chondrocyte apoptosis through PI3K/Akt signaling. NT3, a multifunctional protein with high affinity for TrkC, effectively protects against cartilage degeneration in OA models *in vitro* and *in vivo* and relieves pain sensitivity in mice with OA. Our results indicate that TrkC is crucial for maintaining cartilage homeostasis and OA progression. Targeting TrkC with NT3 could be a novel strategy for OA treatment.

## Introduction

Osteoarthritis (OA) is the most prevalent chronic degenerative joint disease and one of the leading causes of dysfunction and disability among older adults worldwide, and this imposes a heavy financial burden on society 1, 2. OA is characterized by cartilage destruction, synovial inflammation, subchondral bone remodeling, and osteophyte formation. Progressive degeneration of articular cartilage is regarded as the major hallmark of OA 3. Articular cartilage is commonly known as a physiologically poor self-repairing avascular and uninnervated tissue comprising chondrocytes and an extracellular matrix (ECM)^4^. Despite lack of nervous innervation, cartilage metabolism is modulated and influenced by peripheral joint innervation. Changes in peripheral joint innervation are supposed to be partly responsible for degenerative OA. As a unique cell type within the articular cartilage, chondrocytes are responsible for ECM synthesis and secretion to maintain articular cartilage integrity 5-7. Chondrocytes express various receptors for neurotransmitters, allowing response to peripheral neuronal stimuli, such as distinct subtypes of adrenoceptors (AR), receptors for vasoactive intestinal peptide (VIP), substance P (SP) and calcitonin gene-related peptide (CGRP) 8. However, how neurotransmitters regulate chondrocyte homeostasis and the underlying molecular mechanisms remain unclear.

Tropomyosin receptor kinase C (TrkC), a member of the neurotrophic factor family of receptors, is widely expressed in the nervous system and activated by neurotrophin 3 (NT3), and this has an important role in maintaining neuronal survival, promoting neuronal growth and differentiation, and facilitating the repair of damaged neurons 9-12. In addition to its crucial role in the nervous system, accumulating evidence suggests that NT3/TrkC exerts multiple regulatory effect on non-neurological and tumor tissues 13-15. Moreover, it has been implicated in the regulation of angiogenesis and mitogenic activities that promote tumor growth, prevent apoptosis, and facilitate the metastasis and invasion of multiple types of cancers 16. NT3/TrkC expression is also elevated in fractured and injured growth plates 17, 18, and it promotes angiogenesis, chondrogenesis, and osteogenesis at the fracture site 19. However, the involvement and function of NT3/TrkC in OA cartilage degeneration and chondrocyte homeostasis remain unknown. Further studies on the mechanisms underlying NT3/TrkC signaling will be of great significance for OA treatment.

In this study, we first found that the reduced expression level of TrkC in chondrocytes was closely associated with OA and aimed to clarify the role of TrkC in OA cartilage degeneration. To this end, we used the inducible Cre-LoxP system to mediate time-specific gene knockout to produce postnatal TrkCfl/fl; Col2a1-CreERT2 mice and performed the intra-articular injection of an adeno-associated virus to overexpress TrkC. We further performed RNA sequencing to investigate the mechanism underlying the role of TrkC in OA chondrocyte metabolic dyshomeostasis. Finally, we investigated the potential therapeutic effects of NT3 on OA progression. Our study provides new insights into the pathogenesis of OA and identifies a potential therapeutic target for OA.

## Materials And Methods

### Protocol for *in*-*vivo* study

Animal experiments were performed with the approval of the Laboratory Animal Ethics Committee of the Ninth People's Hospital Affiliated to Shanghai Jiao Tong University School of Medicine (SH9H-2023-A80-1). TrkCfl/fl mice were purchased from GemPharmatech Co. Ltd. (Jiangsu, China). TrkCfl/fl mice were mated with Col2a1-CreERT2 mice to generate TrkCfl/fl; Col2a1-CreERT2 mice. Eight-week-old male TrkCfl/fl; Col2a1-CreERT2 mice and their TrkCfl/fl littermates were injected intraperitoneally with tamoxifen (Sigma-Aldrich, St. Louis, MO, USA) at a dosage of 100 μg/g body weight daily for 5 days before the DMM surgery.

Male C57BL/6J mice were obtained from Shanghai Jihui Laboratory Animal Breeding Co. (Shanghai, China) and randomLy divided into experimental groups. All animals were raised under ambient temperature (22-24 °C), constant humidity (40-60%), and a 12-h light/dark cycle with free access to normal food and water. To overexpress TrkC *in vivo*, mice were intra-articularly injected with the AAV. Recombinant AAV2/5 (rAAV2/5) vectors encoding either green fluorescent protein (GFP) alone (rAAV2/5-GFP) or TrkC-GFP (rAAV2/5-TrkC-GFP) were generated by OBiO Technology Corp. Ltd. (Shanghai, China). A volume of 10 μl of AAV-TrkC (1 × 1012 v.g. per mL) was injected into the knee joints using micro-injection needles. To determine the treatment effect of NT3 *in vivo*, mice were injected with 10 μl of recombinant NT3 (50 μg/mL; R&D Systems, Minnesota, USA) or the equivalent volume of PBS into the knee joints weekly.

For the DMM-induced OA model, medial meniscotibial ligament transection was performed. Briefly, the mice were anesthetized, and the surgical site of the knee joint was prepared and sterilized. The joint capsule near the medial patellar tendon was incised, and the medial meniscotibial ligament was excised after exposing the intercondylar region. The skin and joint capsules were then sutured. In the sham-operated group, only the medial meniscotibial ligament was exposed without cutting. The mice were euthanized 8 weeks postoperatively, and knee joint specimens were collected and fixed in 4% PFA for subsequent analysis.

### Micro-CT scanning

Micro-CT (Bruker SkyScan 1072, Kontich, Belgium) was used to examine changes in osteophytes and subchondral cancellous bone in the mouse knee joints. Briefly, knee joints were fixed with 4% PFA for 48 h, washed with running water, and then transferred to 70% ethanol. The specimens were loaded into scanning tubes and scanned (voltage: 55 kV, current: 145 μAmp, resolution: 9 μm). Three-dimensional images of the knee joints were reconstructed, and the microstructural parameters of the subchondral cancellous bone, the including bone volume/total tissue volume, trabecular thickness, and trabecular separation, were calculated, as described in the previous studies 20, 21.

### Histological analysis

Knee joint specimens were decalcified in a 10% EDTA solution (Servicebio, Hubei, China). After decalcification, samples were dehydrated in serially graded ethanol and incubated in xylene. Then the specimens were embedded in paraffin and sectioned serially (4 μm thick). Safranine O /Fast Green (Servicebio) staining was performed to assess cartilage degeneration. The Osteoarthritis Research Society International (OARSI) scoring was determined according to Sophocleous standards 22. H&E (Servicebio) staining was performed to evaluate synovial inflammation. The synovitis score was determined according to the Krenn criteria 23. For each sample, we analyzed three levels of each section (50-μm apart) through the medial compartment of the knee. The scores to indicate the cartilage degeneration and synovial inflammation were evaluated by two observers under blinded conditions. Images of the sections were captured using an optical microscope (Olympus, Japan).

### Immunohistochemical analysis

For immunohistochemistry (IHC) staining, the sections were incubated with a primary antibody against Col2a1 (diluted 1:100) and MMP13 (diluted 1:100) at 4 ℃ overnight. Following primary antibody incubation, a corresponding secondary antibody (Anti-rabbit IgG, diluted 1:1000) conjugated with horseradish peroxidase (HRP) was added to the sections, followed by 3,3'-diaminobenzidine (DAB) staining (Servicebio). Images were captured using an optical microscope (Olympus), and quantitative analysis was performed using the ImageJ software.

### Animal behavioral tests

Mechanical allodynia (von Frey sensitivity) was tested by using a calibrated set of von Frey filaments (Xinruan Technology, Chengdu, China). Before the von Frey test, mice were allowed to adapt to the elevated grid platform for 15 min. A calibrated set of von Frey filaments was applied from below to the plantar aspect of the hind paw to calculate the 50% force withdrawal threshold using an iterative method. Testing for thermal allodynia was performed using a hotplate pain meter (Taimeng, Chengdu, China) at 55 ℃. The response time was defined as the latency period for limb responses, such as paw shaking, licking, or jumping. The tests were performed in a blinded manner, and the same investigator was blinded to the identification of the animals and study groups.

### Human articular cartilage tissue samples

Human OA articular cartilage specimens were obtained from patients with OA undergoing total knee arthroplasty at the Ninth People's Hospital Affiliated to Shanghai Jiao Tong University School of Medicine (n = 19; age, 71.37 ± 7.69 years; 5 males and 14 females). The collection of cartilage samples was approved by the Ethics Committee of the Ninth People's Hospital Affiliated to Shanghai Jiao Tong University School of Medicine. Written informed consent was obtained from all patients involved in this study. Specimens that included all the cartilage layers and subchondral bone were harvested from the medial or lateral parts of the tibial plateau by drilling holes (5 mm) and were fixed in 4% paraformaldehyde (PFA) for subsequent analysis.

### Cell culture

Primary chondrocytes were isolated from the articular cartilage of newborn mice. The articular cartilage samples were cut into pieces and digested with trypsin (Gibco, Carlsbad, CA, USA) for 30 min and 0.2% collagenase type II (Sigma-Aldrich, St Louis, MO, USA) for 6 h at 37 °C. The released primary chondrocytes were incubated and expanded in DMEM/F12 (HyClone, Logan, UT, USA) containing 10% fetal bovine serum (FBS; Gibco) and 1% penicillin-streptomycin solution (Gibco) at 37 °C and with 5% CO2 in a humidified atmosphere. The mouse chondrogenic cell line ATDC5 was purchased from the Cell Bank of the Chinese Academy of Sciences (Shanghai, China) and cultured in DMEM (Hyclone) supplemented with 5% FBS and a 1% penicillin-streptomycin solution.

### RNA sequencing

Total RNA was extracted from (1) primary chondrocytes treated with or without TNFα (10 ng/mL, Sangon Biotech, Shanghai, China), (2) primary chondrocytes from WT and TrkC-cKO mice, and (3) ATDC5 cells transfected with sh-NC or sh-TrkC lentivirus. RNA sequencing was performed by BGI Co. Ltd. (Wuhan, China). The fold-change (up- and downregulated; ≥2.0) was used to identify DEGs, and P-values (≤0.05) were calculated with a t-test. GO, KEGG, and GSEA were used to determine the biological functions and pathways associated with these DEGs.

### Lentivirus transduction

The TrkC sequence was inserted into a lentivector-transferred plasmid to generate a TrkC-overexpressing lentiviral vector (pcSLenti-EF1-EGFP-P2A-Puro-CMV-TrkC-3xFLAG-WPRE), and TrkC short hairpin RNA (shRNA, pSLenti-U6-shRNA (TrkC)-CMV-EGFP-F2A-Puro-WPRE) was synthesized and constructed by OBiO Technology Corp., Ltd. (Shanghai, China). Transfection was performed according to the manufacturer's instructions. Briefly, ATDC5 cells were seeded in a 6-well plate at a density of 1.5 × 105 cells/well. Upon reaching 30-40% confluence, the cells were infected with lentiviruses in the presence of polybrene (10 μg/mL) for 12 h. After 48 h, the infected cells were selected with 3 μg/mL puromycin (Biosharp, Hefei, China). The cellular fluorescence intensity was observed using a fluorescence microscope (Olympus) to assess the infection efficiency.

### Micromass culture

Chondrocytes were digested, centrifuged, and resuspended at a density of 1.5 × 107 cells/mL. A droplet of a 10 μL cell suspension was seeded into the center of a 24-well plate and incubated at 37 ℃ for 2 h, and then, 500 μL of DMEM (Hyclone) was gently added. The medium was changed every other day and the cells were incubated for 7 days. Next, the cells were stained with alcian blue and toluidine blue solutions (Solarbio, Beijing, China) according to the manufacturer's instructions. ECM deposition was quantified using ImageJ software.

### Quantitative real-time PCR

Total RNA was extracted from the cells using an HP Total RNA Kit (Omega Bio-Tek Inc., Norcross, GA, USA). The concentration was measured with a NanoDrop spectrophotometer (Thermo Fisher Scientific, Waltham, MA, USA). RNA was reverse-transcribed into cDNA using the qScript cDNA Synthesis Kit (Takara, Shiga, Japan) according to the manufacturer's instructions. Quantitative real-time PCR was performed using the SYBR Premix Ex Taq Kit (Takara) according to the manufacturer's instructions. Gene expression was normalized to that of the housekeeping gene GAPDH. The primers were purchased from Sangon Biotech (Shanghai, China), and the primer sequences are listed in Table S1.

### Western blotting

The cells were gently washed three times with pre-cooled PBS and lysed with RIPA buffer (Beyotime) supplemented with 1% protease and phosphatase inhibitors (Thermo Fisher Scientific) for 30 min on ice. The lysate supernatant was collected after centrifuging at 12,000 ×g for 15 min at 4 °C. A bicinchoninic acid protein assay kit (Beyotime) was used to quantify the protein concentrations. The samples were boiled at 95 °C for 15 min in a loading buffer. Equal amounts of protein were separated via 4-20% sodium dodecyl sulfate-polyacrylamide gel electrophoresis (GenScript, Nanjing, China) and electro-transferred onto polyvinylidene fluoride membranes (Merck, Kenilworth, New Jersey, USA). Subsequently, the membranes were blocked in 3% bovine serum albumin (Biofroxx, Germany) solution for 1 h at room temperature and incubated overnight with primary antibodies at 4 ℃. Next, the membranes were washed with Tris-buffered saline containing Tween 20 (Epizyme, Shanghai, China) and incubated with a secondary antibody for 1 h in the dark on a shaker. Images were captured using a dual-color infrared laser imaging system (Odyssey, LI-COR Biosciences, Lincoln, NE, USA). Detailed information on the antibodies used in this study is presented in Table S2.

### TUNEL assay

The TUNEL assay was performed using the One Step TUNEL Apoptosis Assay Kit (Beyotime), using the chondrocytes and deparaffinized sections of cartilage, according to the manufacturer's instruction. After TUNEL staining, the nuclei were stained with DAPI (Beyotime) for 15 min in the dark. Images were acquired using a laser scanning confocal microscope (LSCM, TCS SP8; Leica, Germany).

### Statistical analysis

Data from at least three independent experiments using biological replicated samples were presented as the mean ± standard deviation. Statistical analyses were performed using GraphPad Prism software (version 8.0). Statistical comparisons were performed using the unpaired two-tailed Student's t-test between two groups and one-way analysis of variance (ANOVA) followed by Tukey's post-hoc test for more than two groups. Data quantified based on ordinal grading systems including the OARSI grade and the synovial score, whose data points are not continuous and do not follow a normal distribution, were analyzed using non-parametric statistical methods. To determine statistically significant differences, a nonparametric test based on Mann-Whitney U test was used. For non-parametric analysis in multigroup comparisons, Kruskal-Wallis test followed by Mann-Whitney U test was used. Statistical significance was set at P < 0.05.

## Results

### TrkC expression is downregulated in the articular cartilage of patients and mice with OA

To identify differentially expressed genes (DEGs) related to OA pathogenesis, we performed RNA-sequencing analysis of primary chondrocytes, with or without pro-inflammatory cytokine (TNF-α, 10 ng/mL) treatment, comprising a conventional cell model to mimic the destructive environment of progressive OA. After TNF-α treatment, the mRNA expression levels of Col2a1, Comp, Acan, Sox6, and Sox9 were all significantly downregulated, whereas the expression levels of Mmp3, Mmp9, Mmp13, and Adamts5 were upregulated (Figure S1A), similar to the gene expression patterns observed in OA-associated cartilage. Gene Ontology (GO) and Kyoto Encyclopedia of Genes and Genomes (KEGG) pathway analyses of the functions of the DEGs were performed. The terms ECM organization, negative regulation of cell proliferation, apoptotic process, and neurotrophin signaling pathway were significantly enriched (Figure S1B, C).

**Figure 1:**
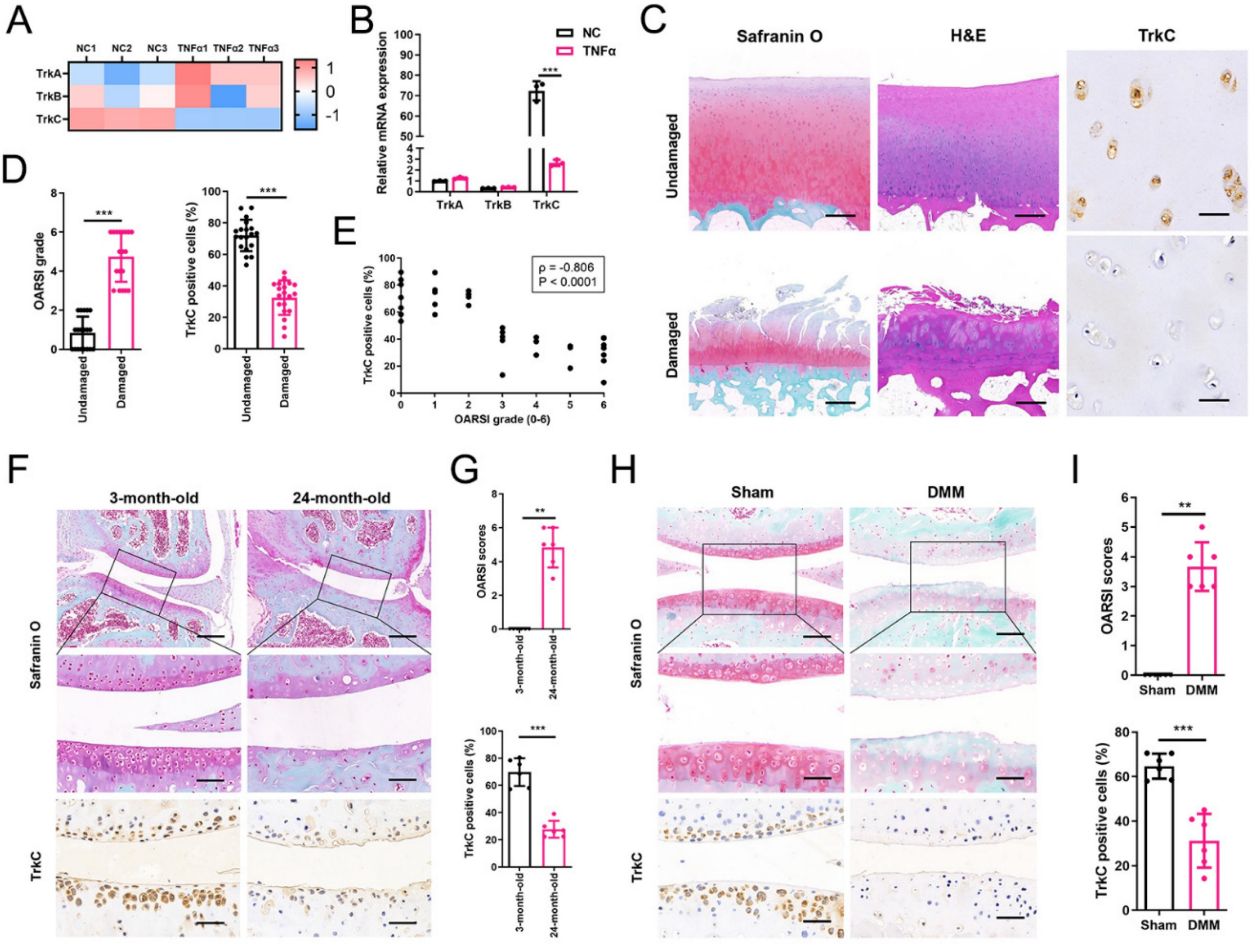
TrkC expression is downregulated in a chondrocytes of an osteoarthritis (OA) model and the articular cartilage of patients and mice with OA. (A) The heat map of neurotrophin receptors in chondrocytes, with or without TNFα treatment. (B) Gene expression of TrkA, TrkB, and TrkC in chondrocytes induced with TNFα verified through PCR. (C) Safranin O-fast green, H&E (scale bar: 200 μm), and TrkC immunohistochemical staining (scale bar: 30 μm) of articular cartilage samples from patients with OA. (D) Statistics of Osteoarthritis Research Society International (OARSI) scores and the percentages of TrkC-positive cells in articular cartilage. (E) Correlation analysis between the proportion of TrkC-positive cells and OARSI score. (F) Safranin O-fast green staining (upper: scale bar: 200 μm; lower: scale bar: 50 μm) and TrkC immunohistochemical staining (scale bar: 20 μm) of articular cartilage of young and aging mice. (G) OARSI scores and percentage of TrkC-positive cells in the articular cartilage of young and aging mice. (H) Safranin O-fast green staining (upper: scale bar: 100 μm; lower: scale bar: 50 μm) and TrkC immunohistochemical staining (scale bar: 20 μm) of articular cartilage, comparing the sham-operated and destabilization of the medial meniscus (DMM) groups. (I) OARSI scores and proportion of TrkC-positive cells in articular cartilage, comparing the sham-operated and DMM groups. *P < 0.05, **P < 0.01, ***P < 0.001.

Analysis of DEGs revealed that TrkC levels were significantly decreased, whereas those of TrkA and TrkB were not (Figure 1A, B). Moreover, TrkC, an important receptor in the neurotrophin signaling pathway, was determined to be closely related to the regulation of cell survival, proliferation, and apoptosis, which is consistent with the results of GO and KEGG analyses. Therefore, we hypothesized that TrkC might be involved and play a vital role in the pathogenesis of OA. PCR and western blotting (WB) further confirmed that TrkC expression was significantly decreased in chondrocytes after treatment with TNFα (Figure S1D, E). TrkC expression was also markedly downregulated in human OA-associated cartilage (Figure 1C, D, Figure S2). TrkC was robustly expressed in undamaged regions but was barely detectable in damaged regions of human OA-associated cartilage. Moreover, its expression showed a strong negative correlation with the Osteoarthritis Research Society International (OARSI) grade based on Spearman's rank correlation coefficient (ρ = -0.806, P < 0.0001; Figure 1E). Similarly, TrkC expression was markedly reduced in the articular cartilage of the aged OA mouse model (Figure 1F, G). We established a destabilization of the medial meniscus (DMM)-induced OA model and found that TrkC expression was substantially reduced in the articular cartilage of mice with OA (Figure 1H, I). Moreover, TrkC expression was decreased during the early stage of DMM-induced OA mice (Figure S3). We also detected the expression of TrkA and TrkB in OA articular cartilage. The results showed that the expression of TrkA and TrkB were both increased in articular cartilage from patients with OA, aging mice, and DMM-induced OA mice (Figure S4-6). Our results suggest that reduced chondrocyte TrkC expression is strongly associated with OA pathogenesis.

### TrkC deletion in chondrocytes exacerbates instability-induced OA progression

**Figure 2:**
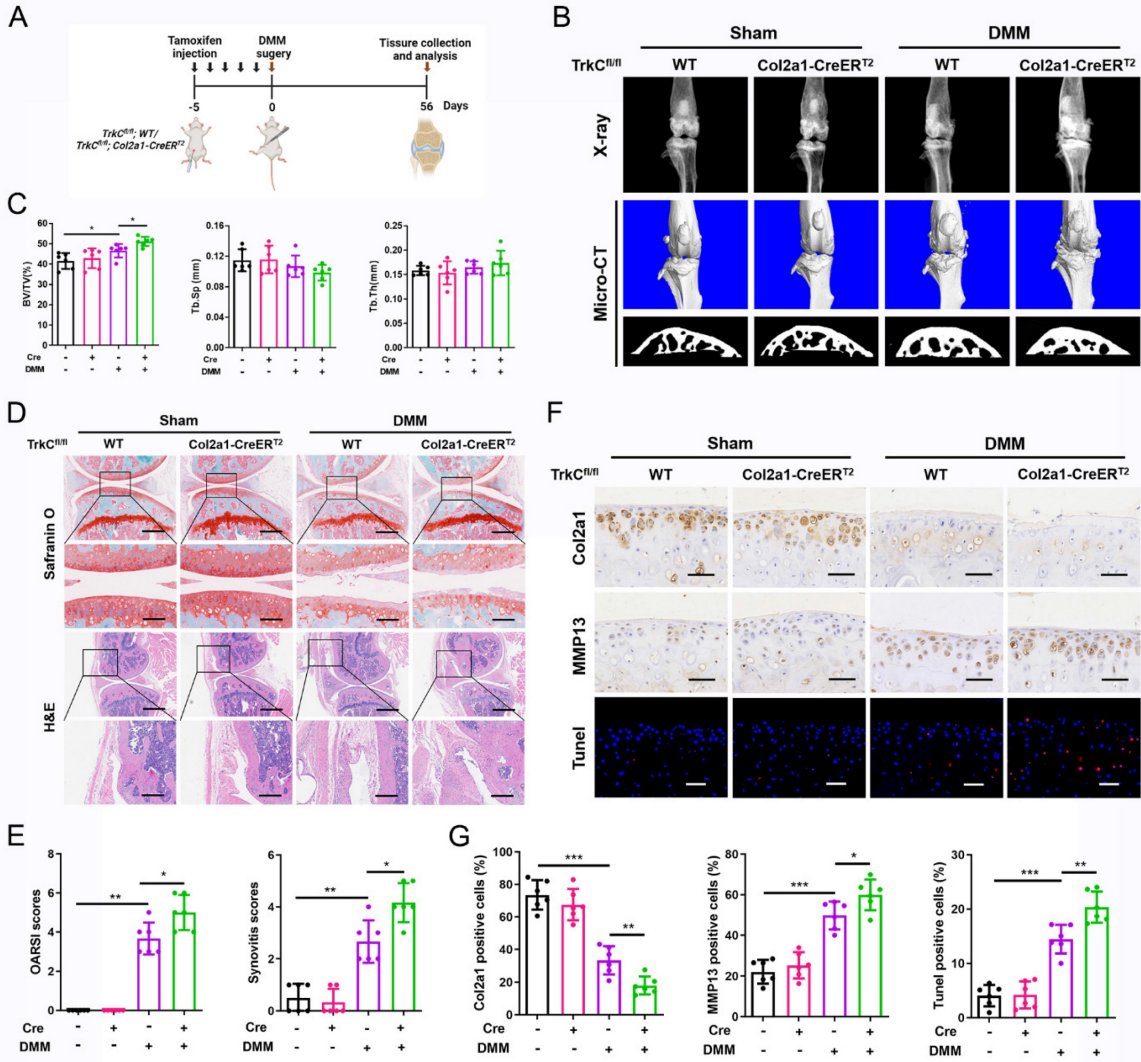
Conditional knockout of TrkC in chondrocytes exacerbates instability-induced osteoarthritis (OA) progression. (A) Schematic illustration of posttraumatic OA model with destabilization of the medial meniscus (DMM) surgery in WT and TrkC-cKO (conditional knockout) mice. (B) Radiographs and micro-CT images of knee joints. (C) Quantitative analysis of bone volume/total tissue volume (BV/TV), trabecular separation (Tb.Sp), and trabecular thickness (Tb.Th) of subchondral bone. (D) Safranin O-fast green staining of cartilage (upper: scale bar: 400 μm; lower: scale bar: 100 μm) and H&E staining of synovium (upper: scale bar: 600 μm; lower: scale bar: 200 μm). (E) Osteoarthritis Research Society International (OARSI) and synovial inflammation scores. (F, G) Immunohistochemical staining (Col2a1 and MMP13) (scale bar: 50 μm) and TUNEL fluorescence staining (scale bar: 50 μm) of articular cartilage. *P < 0.05, **P < 0.01, ***P < 0.001.

To investigate the function of TrkC in OA development and progression, we generated TrkCfl/fl; Col2a1-CreERT2 inducible conditional knockout (cKO) mice and performed DMM surgery, comprising a well-established animal OA model (Figures 2A and S7A, B). The TrkC knockout efficiency was confirmed via immunohistochemical staining (Figure S7C). X-ray and micro-CT scanning showed growing osteophyte formation in TrkC-cKO mice compared to observations in WT mice (TrkCfl/fl littermates) following DMM surgery (Figure 2B). Microstructural parameters of the subchondral trabecular bone revealed remarkable sclerosis during OA-associated bone remodeling in TrkC-cKO mice (Figure 2B, C). Further histological analysis demonstrated that articular cartilage degeneration was more severe in TrkC-cKO mice, accompanied by aggravated synovial hyperplasia, than in the control group that was subjected to DMM surgery (Figure 2D, E). Collectively, TrkC deletion in chondrocytes augmented the DMM-induced OA phenotype, with aggravated cartilage destruction, subchondral bone sclerosis, osteophyte maturation, and synovial inflammation.

To further explore the role of TrkC in articular cartilage homeostasis, we measured the expression of cartilage anabolism- and catabolism-related proteins using immunohistochemistry. Col2a1 expression was distinctly decreased and MMP13 expression was remarkably increased in the articular cartilage of TrkC-cKO mice after DMM surgery, indicating an imbalance in cartilage homeostasis (Figure 2F, G). In addition, chondrocyte apoptosis was significantly increased in TrkC-cKO mice after DMM (Figure 2F, G). These results indicate that TrkC deficiency promotes the development and progression of OA by disrupting articular cartilage homeostasis.

### TrkC overexpression attenuates experimental OA development in mice

**Figure 3:**
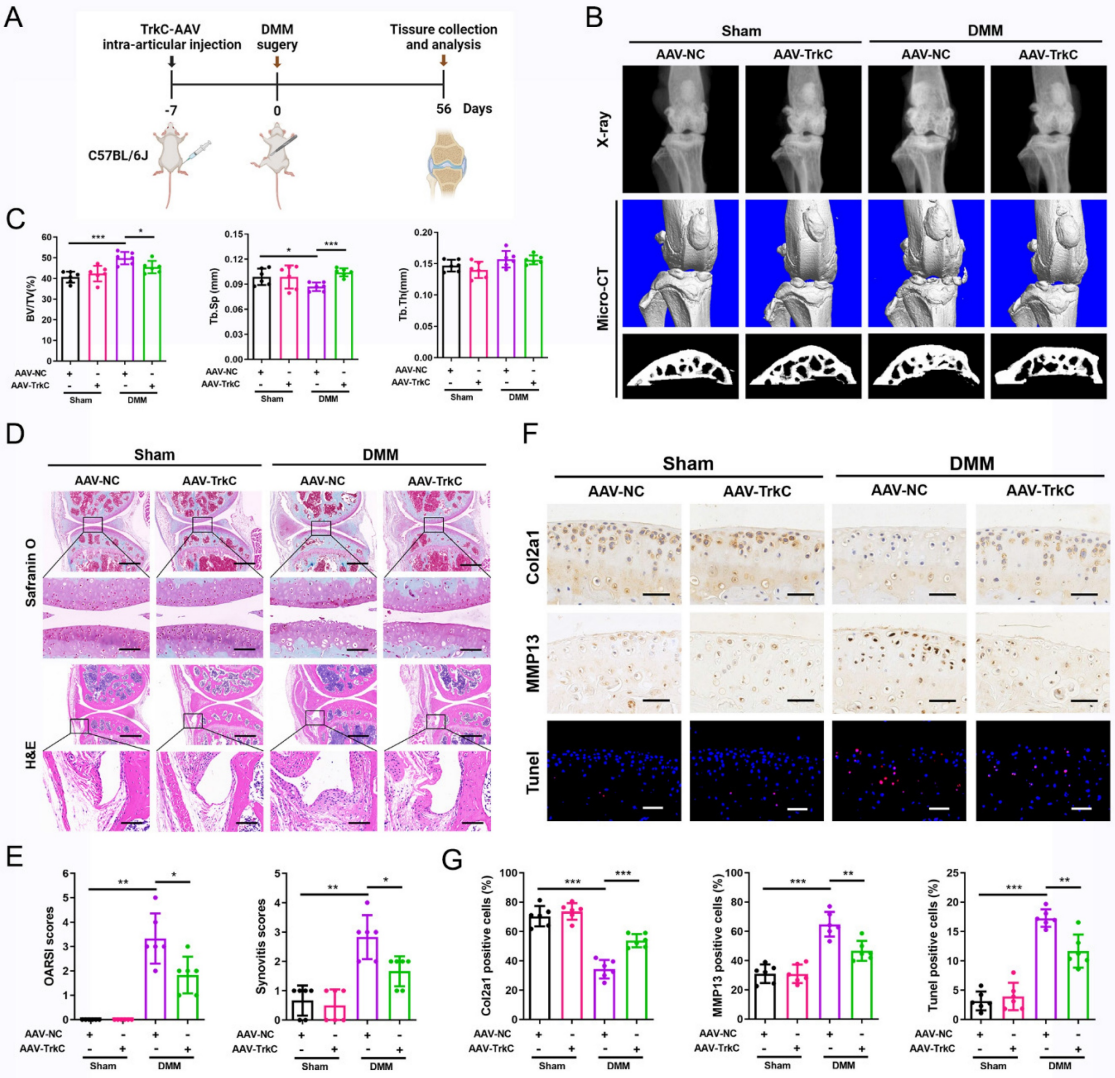
Overexpression of TrkC attenuates the development of experimental osteoarthritis (OA) in mice. (A) Schematic illustration of posttraumatic OA model with destabilization of the medial meniscus (DMM) surgery in adeno-associated virus (AAV)-NC and AAV-TrkC groups. (B) Radiographs and micro-CT images of knee joints. (C) Quantitative analysis of bone volume/total tissue volume (BV/TV), trabecular separation (Tb.Sp), and trabecular thickness (Tb.Th) of subchondral bone. (D) Safranin O-fast green staining of cartilage (upper: scale bar: 400 μm; lower: scale bar: 100 μm) and H&E staining of synovium (upper: scale bar: 600 μm; lower: scale bar: 200 μm). (E) Osteoarthritis Research Society International (OARSI) and synovial inflammation scores. (F, G) Immunohistochemical staining (Col2a1 and MMP13) (scale bar: 50 μm) and TUNEL fluorescence staining (scale bar: 50 μm) of articular cartilage. *P < 0.05, **P < 0.01, ***P < 0.001.

To determine the role of upregulated TrkC expression in OA pathogenesis, a TrkC-overexpressing adeno-associated virus (AAV-TrkC) was injected into the articular cavity of WT mice (Figure 3A). The efficiency of TrkC overexpression in articular cartilage was examined using immunohistochemical staining (Figure S8). The radiographic results demonstrated that TrkC overexpression significantly reduced DMM-induced osteophyte formation (Figure 3B). Histomorphometric analyses showed that subchondral osteosclerosis was alleviated in the AAV-TrkC+DMM group compared to that in the AAV-NC+DMM group (Figure 3B, C). Histological analysis of Safranin O and H&E staining revealed that TrkC overexpression resulted in retention of the general integrity of articular cartilage and relieved synovial hyperplasia, with lower OARSI and synovitis scores (Figure 3D, E). Immunohistochemistry showed that the expression of Col2a1 was significantly decreased and that of MMP13 was markedly elevated after DMM surgery; however, TrkC overexpression partially reversed the expression of these anabolic and catabolic markers (Figure 3F, G). In addition, chondrocyte apoptosis was significantly ameliorated in the AAV-TrkC+DMM group compared to that in the AAV-NC+DMM group (Figure 3F, G). Thus, our data suggest that upregulated TrkC expression delays OA development and progression and preserves the integrity of articular cartilage.

### TrkC deficiency disturbs chondrocyte homeostasis

**Figure 4:**
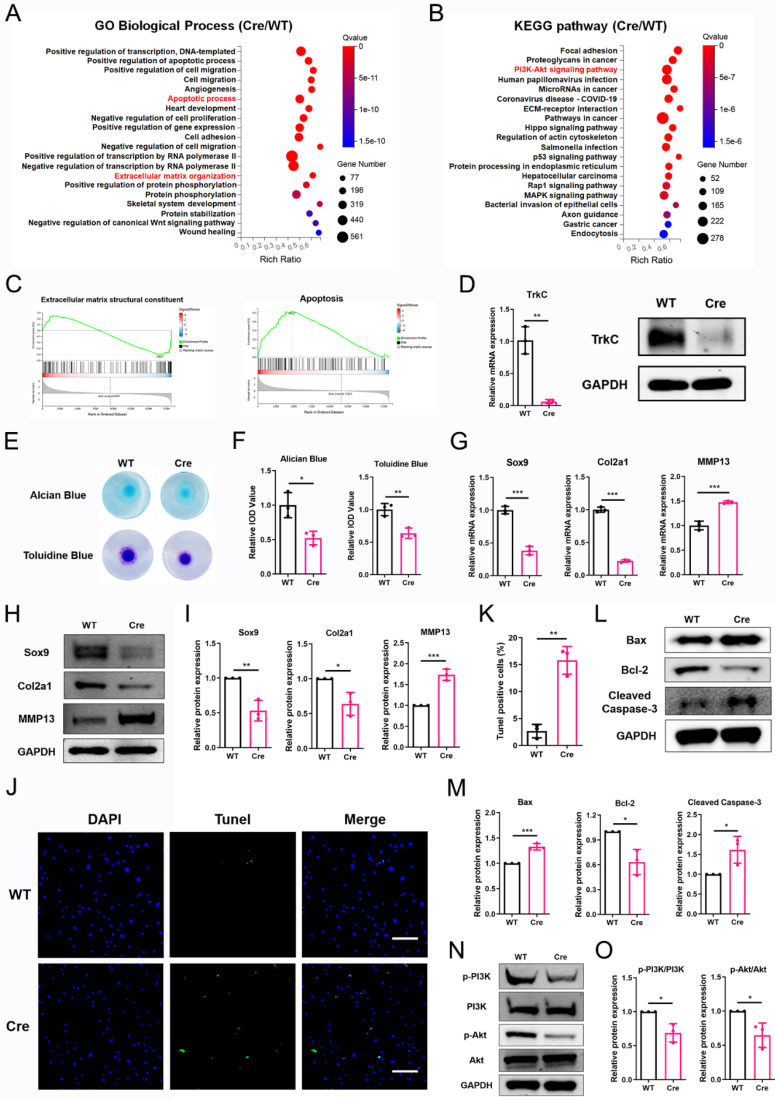
TrkC deficiency disturbs chondrocytes homeostasis. (A, B) Gene Ontology (GO) and Kyoto Encyclopedia of Genes and Genomes (KEGG) enrichment analysis of differentially expressed genes between control and TrkC-knockout chondrocytes. (C) Gene set enrichment analysis. (D) Knockout efficiency of TrkC, verified through PCR and WB. (E, F) Alcian blue and toluidine blue staining. (G) mRNA expression of chondrocytes extracellular matrix metabolism marker genes after TrkC knockout. (H, I) Expression of chondrocyte extracellular matrix metabolism-related proteins after TrkC knockout. (J, K) TUNEL fluorescence comparing WT and TrkC-knockout chondrocytes. Scale bar: 100 μm. (L, M) Expression of chondrocyte apoptosis-related proteins after TrkC knockout. (N, O) TrkC knockout decreased the protein expression of p-PI3K and p-Akt (Ser473). *P < 0.05, **P < 0.01, ***P < 0.001.

To investigate the mechanism underlying the effect of TrkC in OA, we performed RNA sequencing of primary chondrocytes from TrkC-cKO and WT mice. A volcano plot identified 7076 DEGs, comprising 3680 upregulated and 3396 downregulated genes (Figure S9A). Of them DEGs, the upregulated genes included catabolic marker genes, such as MMP3, ADAMTS1, and ADAMTS5, whereas the downregulated genes included anabolic marker genes including Col2a1 and COMP (Figure S9B). GO and KEGG pathway analyses indicated that the apoptotic process, ECM organization, and PI3K/Akt signaling pathway were significantly enriched (Figure 4A, B). Gene set enrichment analysis (GSEA) showed that downregulated gene sets associated with ECM structural constituents and upregulated gene sets associated with apoptosis were preferentially enriched (Figure 4C), suggesting that TrkC is closely involved in chondrocyte homeostasis.

Based on the RNA-sequencing results, we further investigated the role of TrkC in ECM metabolism and chondrocyte apoptosis. The knockout efficiency was verified (Figure 4D). Micromass culture demonstrated that TrkC deficiency attenuated ECM synthesis (Figure 4E, F). PCR and WB results showed that TrkC deficiency resulted in reduced expression of Sox9 and Col2a1 and increased expression of MMP13 (Figure 4G-I). TUNEL staining results demonstrated that TrkC deletion promoted chondrocyte apoptosis (Figure 4J, K). WB results showed that TrkC deletion facilitated expression of the pro-apoptotic proteins Bax and cleaved-caspase 3 and restrained expression of the anti-apoptotic protein Bcl2 (Figure 4L, M). Previous studies have indicated that TrkC phosphorylates tyrosine protein kinases and activates the PI3K/Akt signaling pathway and its downstream targets in cancer cells 24. Furthermore, KEGG analysis revealed that the PI3K/Akt signaling pathway was significantly enriched in TrkC-knockout chondrocytes (Figure 4B). Therefore, we further examined changes in the PI3K/Akt signaling pathway. TrkC deletion significantly suppressed the phosphorylation of PI3K and Akt (Figure 4N, O). Collectively, these results indicate that TrkC might regulate ECM metabolism and chondrocyte apoptosis through the PI3K/Akt signaling pathway.

To clarify the function of TrkC in OA, we used a TrkC-targeting shRNA lentivirus to transfect ATDC5 cells, and similar results were obtained. A volcano plot detected 4025 DEGs, of which 2035 were upregulated and 1990 were downregulated (Figure S10A). GO and KEGG pathway analyses demonstrated that the apoptotic process, ECM organization, chondrocyte differentiation, and PI3K/Akt signaling pathways were significantly enriched (Figure S10B, C). GSEA showed that upregulated gene sets associated with apoptosis and p53 signaling pathway were preferentially enriched (Figure S10D, E). We further investigated the effects of TrkC knockdown on ECM metabolism and chondrocyte apoptosis. The knockdown efficiency was verified (Figure S11A), and alcian blue and toluidine blue staining revealed that TrkC knockdown impaired ECM anabolism (Figure S11B, C). PCR and WB results demonstrated that TrkC knockdown decreased the expression of Sox9 and Col2a1 and increased the expression of MMP13 (Figure S11D-F). Further, TrkC knockdown facilitated chondrocyte apoptosis as shown based on the TUNEL staining results (Figure S12A, B). WB results showed that TrkC knockdown enhanced the expression of Bax, cleaved caspase 3 and cleaved PARP and alleviated the expression of Bcl2 (Figure S12C, D). Moreover, TrkC knockdown significantly suppressed the expression of p-PI3K and p-Akt (Figure S12E, F).

### TrkC overexpression restores ECM metabolism and chondrocyte apoptosis via the PI3K/Akt pathway

**Figure 5:**
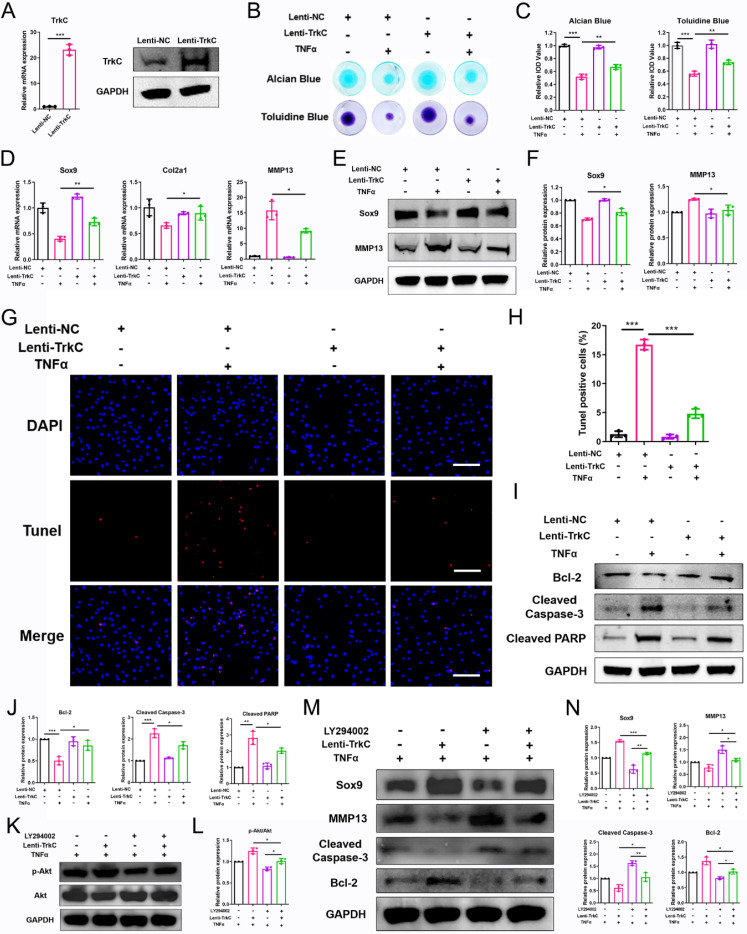
TrkC overexpression restores extracellular matrix metabolism and chondrocyte apoptosis via the PI3K/Akt pathway. (A) Elevated expression of TrkC was verified through PCR and western blotting (WB). (B, C) Alcian blue and toluidine blue staining. (D) mRNA expression of chondrocyte extracellular matrix metabolism marker genes after overexpressing TrkC. (E, F) Expression of chondrocyte extracellular matrix metabolism-related proteins after overexpressing TrkC. (G, H) TUNEL fluorescence staining. Scale bar: 100 μm. (I, J) Expression of chondrocyte apoptosis-related proteins after overexpressing TrkC. (K, L) TrkC overexpression promoted the protein expression of p-Akt (Ser473), and LY294002 addition suppressed the phosphorylation of Akt (Ser473). (M, N) The addition of LY294002 partially reversed the upregulated protein levels of Sox9 and Bcl-2 and the downregulated protein levels of MMP13 and cleaved-caspase 3 induced by TrkC overexpression. *P < 0.05, **P < 0.01, ***P < 0.001.

We next explored the effect of the upregulation of TrkC expression on chondrocyte dyshomeostasis induced by pro-inflammatory cytokines. Here, enhanced TrkC expression was observed (Figure 5A). The results of alcian blue and toluidine blue staining showed that ECM deposition by chondrocytes was significantly reduced after TNFα (10 ng/mL) treatment; however, TrkC overexpression promoted ECM synthesis (Figure 5B, C). PCR and WB results demonstrated that TNFα stimulation decreased the expression of Sox9 and increased the expression of MMP13, but TrkC overexpression reversed the trends of the expression of these anabolic and catabolic markers (Figure 5D-F). Moreover, TrkC overexpression restored TNFα-induced chondrocyte apoptosis based on TUNEL staining (Figure 5G, H). In addition, WB results revealed that TrkC overexpression restrained expression of the pro-apoptotic proteins cleaved-caspase 3 and cleaved-PARP and enhanced expression of the anti-apoptotic protein Bcl2 (Figure 5I, J).

Next, a PI3K-Akt pathway inhibitor (20 μM LY294002) was used to determine whether TrkC overexpression could restore ECM catabolism and chondrocyte apoptosis via PI3K/Akt signaling. WB results demonstrated that LY294002 suppressed phosphorylation of the PI3K-Akt signaling pathway components (Figure 5K, L). Consistent with the decreased expression of p-AKT, the upregulated protein expression of Sox9 and Bcl2 and the downregulated protein expression of MMP13 and cleaved-caspase 3 in the TrkC-overexpressing group were partially reversed by this signaling inhibitor (Figure 5M, N). Overall, our results indicate that TrkC overexpression can rescue ECM degradation and chondrocyte apoptosis via the PI3K/Akt pathway.

### NT3 treatment protects chondrocyte homeostasis and prevents OA progression *in vitro* and *in vivo*

**Figure 6:**
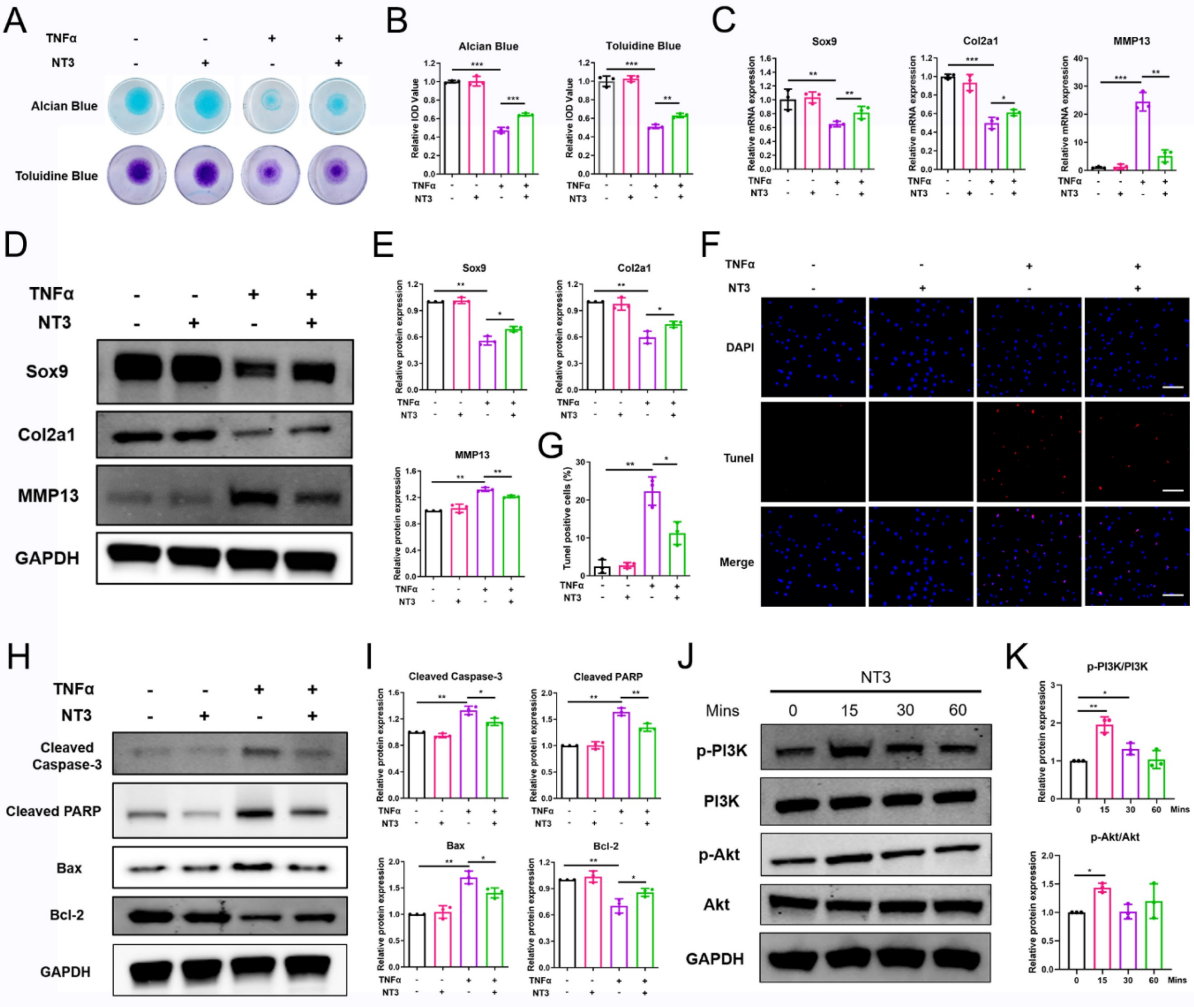
NT3 rescues TNFα-induced extracellular matrix breakdown and chondrocyte apoptosis *in vitro*. (A, B) Alcian blue and toluidine blue staining. (C) mRNA expression of chondrocyte extracellular matrix metabolism-marker genes after NT3 treatment. (D, E) Expression of chondrocyte extracellular matrix metabolism-related proteins after NT3 treatment. (F, G) TUNEL fluorescence staining. Scale bar: 100 μm. (H, I) Expression of chondrocyte apoptosis-related proteins after NT3 treatment. (J, K) NT3 activated the phosphorylation of PI3K and Akt (Ser473) proteins. *P < 0.05, **P < 0.01, ***P < 0.001.

NT3 is a multifunctional protein with a high affinity for TrkC. To investigate its therapeutic potential in OA, we evaluated its effects on chondrocyte homeostasis and OA progression *in vitro* and *in vivo*. For the *in vitro* experiment, the chondrocyte was treated with recombinant NT3 (100 ng/mL)^18^, ^25^-^27^. Alcian blue and toluidine blue staining showed that recombinant NT3 treatment rescued the ECM degradation induced by TNFα stimulation (Figure 6A, B). PCR and WB results demonstrated that NT3 could promote the expression of chondrocyte anabolic factors (Sox9 and Col2a1) and suppress that of the catabolic factor (MMP13) after TNFα treatment (Figure 6C-E). TUNEL staining results revealed that TNFα treatment facilitated chondrocyte apoptosis; however, NT3 rescued chondrocyte apoptosis (Figure 6F, G). WB analysis also showed that NT3 restrained expression of the pro-apoptotic proteins cleaved-caspase 3, cleaved-PARP, and Bax and enhanced expression of the anti-apoptotic protein Bcl2 (Figure 6H, I). To determine whether NT3 activates the downstream PI3K/Akt signaling pathway by binding to TrkC, we analyzed the protein phosphorylation levels of PI3K and Akt. WB results showed that NT3 treatment induced the phosphorylation of both (Figure 6J, K).

**Figure 7:**
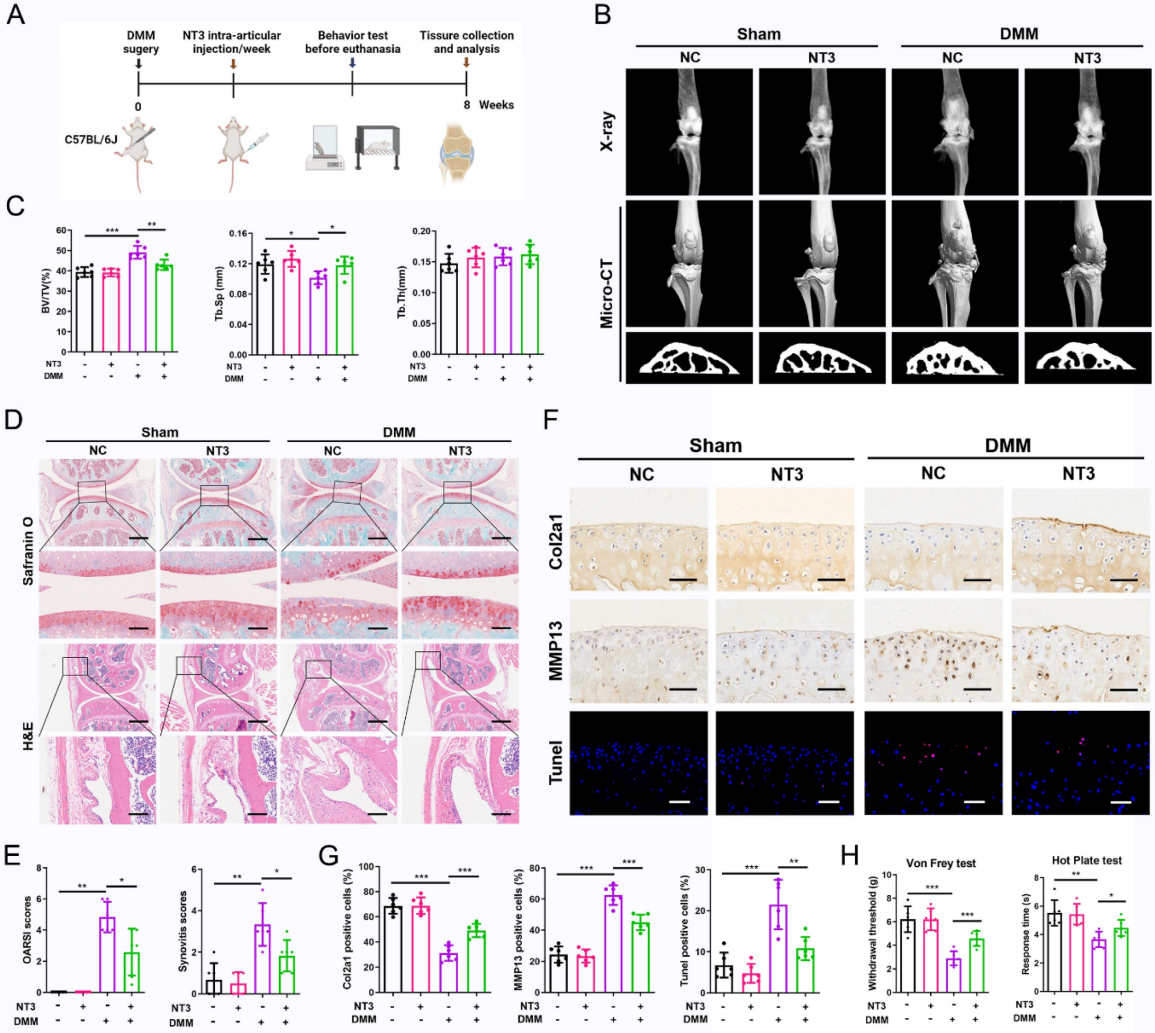
NT3 treatment promotes chondrocytes homeostasis and prevents osteoarthritis (OA) progression *in vivo*. (A) Schematic illustration of destabilization of the medial meniscus (DMM) OA model for NC and NT3 groups. (B) Radiographs and micro-CT images of knee joints. (C) Quantitative analysis of bone volume/total tissue volume (BV/TV), trabecular separation (Tb.Sp), and trabecular thickness (Tb.Th) of subchondral bone. (D) Safranin O-fast green staining of cartilage (upper: scale bar: 400 μm; lower: scale bar: 100 μm) and H&E staining of synovium (upper: scale bar: 600 μm; lower: scale bar: 200 μm). (E) OARSI and synovial inflammation scores. (F, G) Immunohistochemical staining (Col2a1 and MMP13) (scale bar: 50 μm) and TUNEL fluorescence staining (scale bar: 50 μm) of articular cartilage. (H) The von Frey and hot plate tests. *P < 0.05, **P < 0.01, ***P < 0.001.

We further confirmed the therapeutic effects of NT3 on OA progression using a mouse model of DMM-induced OA. The intra-articular injection of recombinant NT3 (50 μg/mL, 10 μl/knee) was implemented once per week for 2 months (Figure 7A). The radiographic analysis showed a significant difference in the knee joint structure of mice in the different treatment groups. Increased osteophyte formation was observed in mice that underwent DMM surgery, but this effect was significantly inhibited by NT3 treatment (Figure 7B). We further analyzed the microstructural changes in the subchondral bone and found that subchondral bone ossification was significantly increased after DMM surgery and that NT3 treatment alleviated DMM-induced subchondral osteosclerosis (Figure 7B, C). Histological analyses demonstrated that cartilage degeneration and synovial hyperplasia were severe following DMM surgery, as evidenced by the increased OARSI and synovitis scores (Figure 7D, E). The administration of NT3 caused a partial but significant reduction in cartilage degradation and synovial hyperplasia (Figure 7D, E). Immunohistochemical staining revealed a significant decrease in Col2a1 expression and an increase in MMP13 expression in the articular cartilage after DMM surgery, which was largely reversed by recombinant NT3 (Figure 7F, G). Moreover, the administration of recombinant NT3 rescued the increase in chondrocyte apoptosis induced by DMM (Figure 7F, G). Collectively, these results suggested that NT3 could retard OA development in a DMM-induced OA mouse model.

We also performed a series of tests to determine whether NT3 could reduce sensitivity to OA pain. The von Frey test showed significantly reduced paw withdrawal response thresholds in mice after DMM surgery. However, the administration of NT3 significantly increased the paw withdrawal response threshold, reflecting reduced pain sensitivity in mice (Figure 7H). We also detected thermal pain sensitivity by performing a hot plate analysis. We found that the response time decreased significantly after DMM surgery and that treatment with NT3 prolonged the response time (Figure 7H). Collectively, these results suggest that NT3 is associated with reduced mechanical and thermal pain sensitivity in mice with OA.

## Discussion

Accumulating evidence indicates that skeletal metabolism is tightly regulated by the nervous system and that skeletal innervation regulates bone homeostasis 28. Unlike the bone, synovium and other musculoskeletal tissues, healthy cartilage does not contain blood vessels and is not innervated by nerve fibers. However, despite lack of nervous innervation, cartilage metabolism is modulated and influenced by neurotransmitters released either from nerve fibers located in neighboring tissue or directly from chondrocytes. Chondrocytes express various receptors for neurotransmitters, allowing response to peripheral neuronal stimuli, such as distinct subtypes of AR, receptors for VIP, SP and CGRP. Opolka et al. demonstrated that neurokinin type 1 (NK-1) receptor, α-AR, and β-AR were abundantly expressed in primary chondrocytes. SP dose dependently promoted chondrocytes proliferation via the NK-1 receptor, whereas NE decreased chondrocytes apoptosis by β-AR 29. Lorenz et al. Showed that NE could reverse IL-1β induced changes in IL-8, MMP-13, glycosaminoglycan, and collagen II expression through β-AR signaling 30.

Neurotrophin and their receptors play a crucial role in maintaining neuronal survival, promoting neuronal proliferation and differentiation, and repair of damaged neurons. The neurotrophic factor family of receptors mainly includes TrkA, TrkB, TrkC, and the low-affinity neurotrophic receptor p75^31^. Trk receptors selectively interact with different neurotrophins, including nerve growth factor (NGF) binding to TrkA, BDNF and NT4 combining with TrkB, and NT3 interacting with TrkC with high affinity 32. Neurotrophin signaling plays important roles in the growth, development, and repair of the nervous system 33. Moreover, the aberrant regulation of these molecules might be associated with the onset and progression of many neurological diseases 34, 35. In addition, increasing number of studies have indicated that neurotrophic receptors are involved in regulating non-neurological tissue, including tumorigenesis and metastasis, as well as skeletal tissue formation and healing 19, 36. However, the involvement and functions of neurotrophic receptors in OA pathogenesis remain unknown. In our study, using RNA sequencing analysis, we firstly found that the expression of TrkC, but not TrkA and TrkB, is significantly decreased in OA-associated chondrocytes. Furthermore, TrkC expression was markedly downregulated in human OA-associated cartilage and various types of OA mouse models. Therefore, we speculate that TrkC might be closely related to and play a vital role in the pathogenesis of OA.

Cartilage homeostasis, defined as the balance between ECM synthesis and degradation, is vital for articular cartilage health 37. The destruction of cartilage homeostasis, marked by the elevated production of MMPs and ADAMTSs and reduced collagen II and aggrecan, is the initiator and promoter of OA pathogenesis 38. Chondrocyte apoptosis can reduce ECM synthesis and secretion and disrupt cartilage homeostasis 39. Previous studies have shown that TrkC influences the survival, proliferation, differentiation, and death of both neuronal and non-neuronal cells. Castellanos et al. reported that TrkC overexpression enhances the survival and migration of neural stem cell transplants in the rat spinal cord 40. Jin et al. found that TrkC is frequently overexpressed in human breast cancer and is a critical regulator of breast cancer cell growth and metastasis 41. However, whether TrkC regulates cartilage homeostasis and chondrocyte apoptosis in OA remains unclear. In this study, we generated inducible TrkC-cKO mice and observed that chondrocyte-specific TrkC deficiency aggravated cartilage destruction. However, the intra-articular injection of TrkC-overexpressing AAV delayed the experimentally induced OA progression. *In vitro*, TrkC deficiency led to decreased anabolic and increased catabolic activities in chondrocytes with the stimulation of chondrocyte apoptosis, whereas TrkC overexpression rescued the imbalance between ECM synthesis and degradation and reduced chondrocyte apoptosis.

The PI3K/Akt signaling pathway is required for cartilage homeostasis and is involved in ECM synthesis 42. Excessive chondrocyte apoptosis is frequently accompanied by cartilage degradation. The PI3K/Akt signaling pathway negatively modulates chondrocyte apoptosis and degradation under multiple pathological conditions, and activated signaling protects against OA by reducing chondrocyte apoptosis and cartilage degradation 43. Li et al. found that a GLP hydrogel protects against IL-1β-induced chondrocyte apoptosis and ECM catabolism by regulating the PI3K/Akt signaling pathway 44. Moreover, Wen et al. reported that miR-455-3p reduces apoptosis and alleviates chondrocyte degeneration by regulating the PI3K/Akt signaling pathway 45. Further, Yao et al. found that fibroblast growth factor 18 (FGF18) attenuates IL-1β-induced chondrocyte apoptosis and cartilage degradation via the PI3K/Akt signaling pathway 46. TrkC mediates many biological functions, such as cell survival, apoptosis, and differentiation, through the PI3K/Akt signaling pathway. Jin et al. found that TrkC induces the PI3K-Akt cascade as a critical regulator of breast cancer cell growth and metastasis 24, 41. In addition, Kim et al. revealed that TrkC significantly inhibits apoptosis by inducing the expression of PLK-1 and Twist-1 through activation of the PI3K/Akt/mTOR pathway 15. However, the mechanism underlying the TrkC-mediated regulation of cartilage homeostasis and chondrocyte apoptosis in OA remains unclear. Whether TrkC mediates these effects via the PI3K/Akt signaling pathway has not yet been investigated. Our study showed that PI3K/Akt signaling pathway was significantly enriched in TrkC-knockout chondrocytes through KEGG pathway analyses. TrkC deficiency significantly suppresses the phosphorylation of PI3K and Akt and that TrkC overexpression increases the expression of PI3K/Akt pathway. However, when we used the PI3K inhibitor LY294002, the restorative effect of TrkC overexpression on ECM degradation and chondrocyte apoptosis was partially reversed. Our results revealed that TrkC regulates ECM metabolism and chondrocyte apoptosis via the PI3K/Akt signaling pathway.

NT3, a multifunctional neurotrophic factor with a high affinity for TrkC, influences the survival, proliferation, differentiation, and death of neuronal and non-neuronal cells. Previous studies have demonstrated that NT3 plays an integral role in the nervous system 47. Moreover, evidence implies that NT3 also has a role in bony repair, which could improve osteogenesis and vascularization after bone injury 18, 48. However, few studies have investigated whether NT3 regulates cartilage homeostasis to protect against OA progression. Our results showed that NT3 treatment activates the TrkC-downstream PI3K/Akt signaling pathway and rescues ECM degradation and chondrocyte apoptosis *in vitro*. Moreover, the intra-articular injection of recombinant NT3 retarded OA development in DMM-induced OA *in vivo*.

Pain is one of the most prominent characteristics of OA, causing disability and economic burden 49, 50. NGF is an important intermediate mediator of OA pain and has been found to be significantly upregulated in the joints of OA patients, which can result in long-lasting mechanical and thermal hyperalgesia 51. NGF inhibitors, including tanezumab, have high selectivity and specificity for binding to NGF to prevent the interaction between NGF and its receptors, thereby disrupting ongoing pain signaling 52, 53. Clinical trials have shown that anti-NGF monoclonal antibody can significantly improve pain relief and functional recovery with knee and hip OA compared to those in the placebo group 54. However, reports have shown that serious joint-related adverse events, such as osteonecrosis and the rapid destruction of joints, are associated with NGF inhibition 55, 56. NT3 belongs to the same family of neurotrophic factors as NGF. However, whether NT3 reduces sensitivity to OA pain has not yet been reported. Our study showed that recombinant NT3 treatment was associated with reduced mechanical and thermal pain sensitivity in OA mice and protected cartilage degeneration simultaneously, which could result in a novel and potential strategy for OA treatment.

**Figure 8:**
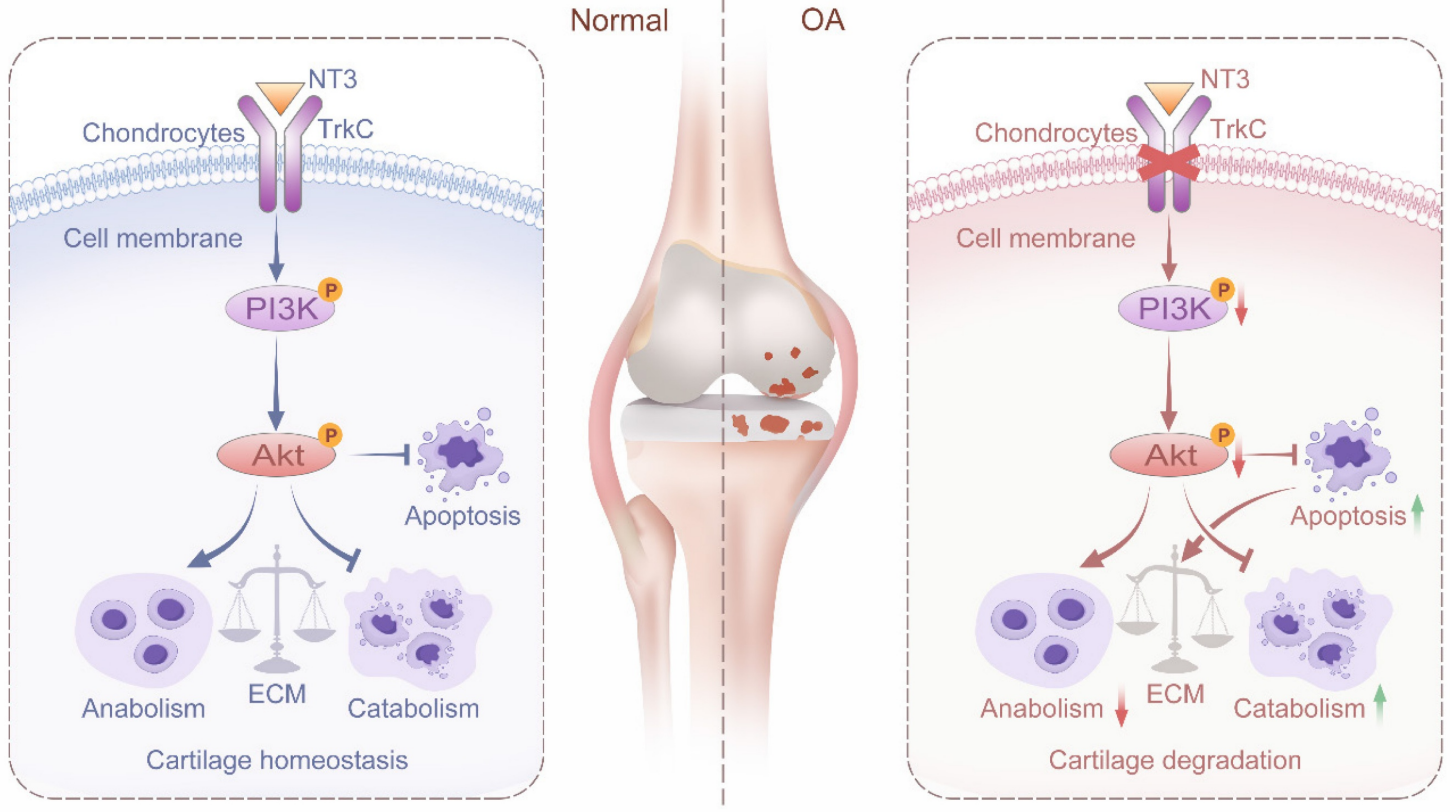
Schematic diagram depicting the role of TrkC in protecting against osteoarthritis progression by maintaining articular cartilage homeostasis and regulating the PI3K/Akt signaling pathway.

In summary, we found that TrkC expression is significantly decreased in OA-associated chondrocytes and in the cartilage of patients and mice with OA. Further, TrkC plays an important role in the regulation of OA. Chondrocyte-specific TrkC deficiency aggravates cartilage destruction in OA-model mice. However, the upregulation of its expression delays DMM-induced OA progression. Furthermore, TrkC influences chondrocyte metabolic homeostasis and apoptosis via the PI3K/Akt signaling pathway. Finally, NT3, the ligand of TrkC, effectively protects against cartilage degeneration and chondrocyte apoptosis in the OA model both *in vitro* and *in vivo* (Figure 8). Our results ultimately indicated that TrkC plays a crucial role in maintaining cartilage homeostasis and OA progression. Targeting TrkC could thus be a novel strategy for the treatment of OA.

## Supplementary Material

Supplementary figures and tables.

## Abbreviations

OA: Osteoarthritis
NT3: Neurotrophin 3
TrkC: Tropomyosin receptor kinase C
ECM: Extracellular matrix
PFA: Paraformaldehyde
DEGs: Differentially expressed genes
GO: Gene Ontology
KEGG: Kyoto Encyclopedia of Genes and Genomes
GSEA: Gene set enrichment analysis
DMM: Destabilization of the medial meniscus
WT: Wild type
cKO: conditional knockout
