# Supplementary material for: TrkC protects against osteoarthritis progression by maintaining articular cartilage homeostasis
Source: Int J Biol Sci. 2025 May 27;21(8):3597–613. doi: 10.7150/ijbs.108832 (PMC12160922; doi:10.7150/ijbs.108832)
Supplement: Supplementary file 1 — Supplementary figures and tables. [file ijbsv21p3597s1.pdf]

Supplementary Information

TrkC protects against osteoarthritis progression by maintaining articular cartilage homeostasis

Yongyun Chang[1], Keyu Kong[1], Hua Qiao[1], Minghao Jin[1], Xinru Wu[1], Wenxuan Fan[1], Jingwei Zhang[1], Yansong Qi[2], Yongsheng Xu[2], An Qin[1*], Zanjing Zhai[1*], Huiwu Li[1*]

[1] Shanghai Key Laboratory of Orthopaedic Implants, Department of Orthopaedics, Ninth People’s Hospital, Shanghai Jiao Tong University School of Medicine, Shanghai, China

[2] Department of Orthopedics, Inner Mongolia People's Hospital, Hohhot, China

These authors have contributed equally to this work: Yongyun Chang, Keyu Kong, Hua Qiao

* Corresponding Authors: An Qin, Zanjing Zhai, Huiwu Li

E-mail addresses: dr_qinan@163.com, zanjing_zhai@163.com, huiwu1223@163.com

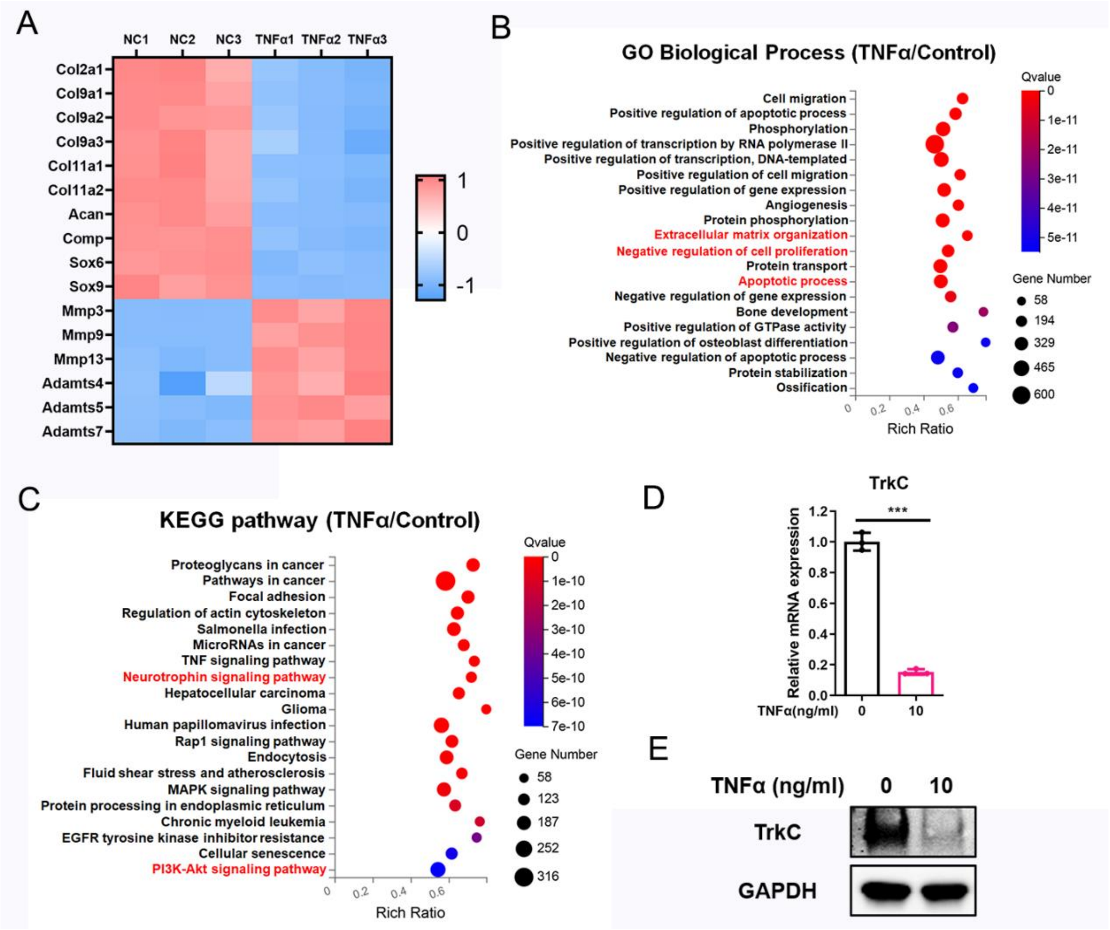

Fig. S1. TrkC expression was downregulated in TNFα-induced chondrocytes. (A) The heatmap of differentially expressed genes between control and TNFα-induced groups. (B, C) Gene Ontology (GO) and Kyoto Encyclopedia of Genes and Genomes (KEGG) enrichment analysis of the differentially expressed genes. (D, E) TNFα treatment reduced the mRNA and protein expression of TrkC. $*P < 0.05$, $**P < 0.01$, $***P < 0.001$.

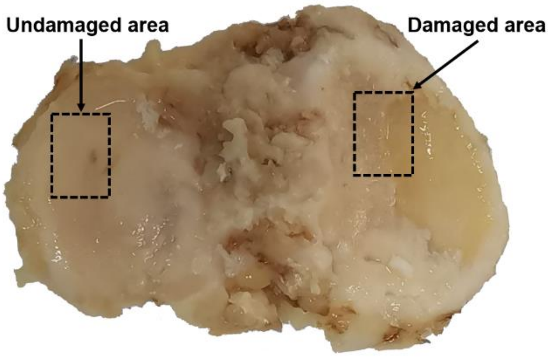

Fig. S2. Human OA knee joint articular cartilage specimens. Black dashed boxes indicate respective undamaged and damaged areas.

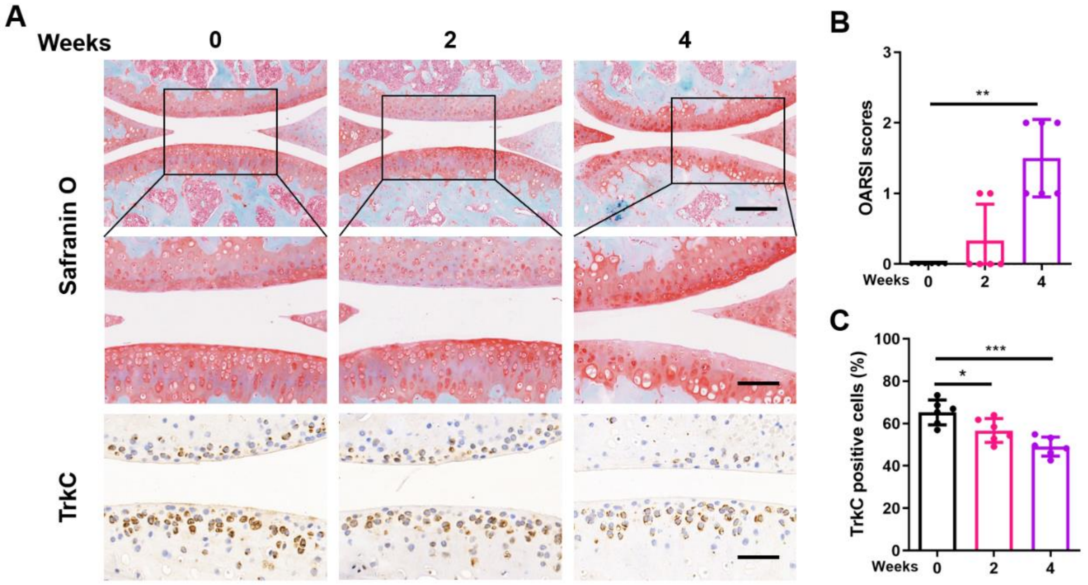

Fig. S3. TrkC expression was downregulated during the early stage of OA. (A) Safranin O-fast green staining (upper: scale bar: 200 μm; lower: scale bar: 100 μm) and TrkC immunohistochemical staining (scale bar: 50 μm) of articular cartilage at 0, 2, 4 weeks after DMM surgery. (B, C) OARSI scores and proportion of TrkC-positive cells in articular cartilage. *P < 0.05, **P < 0.01, ***P < 0.001.

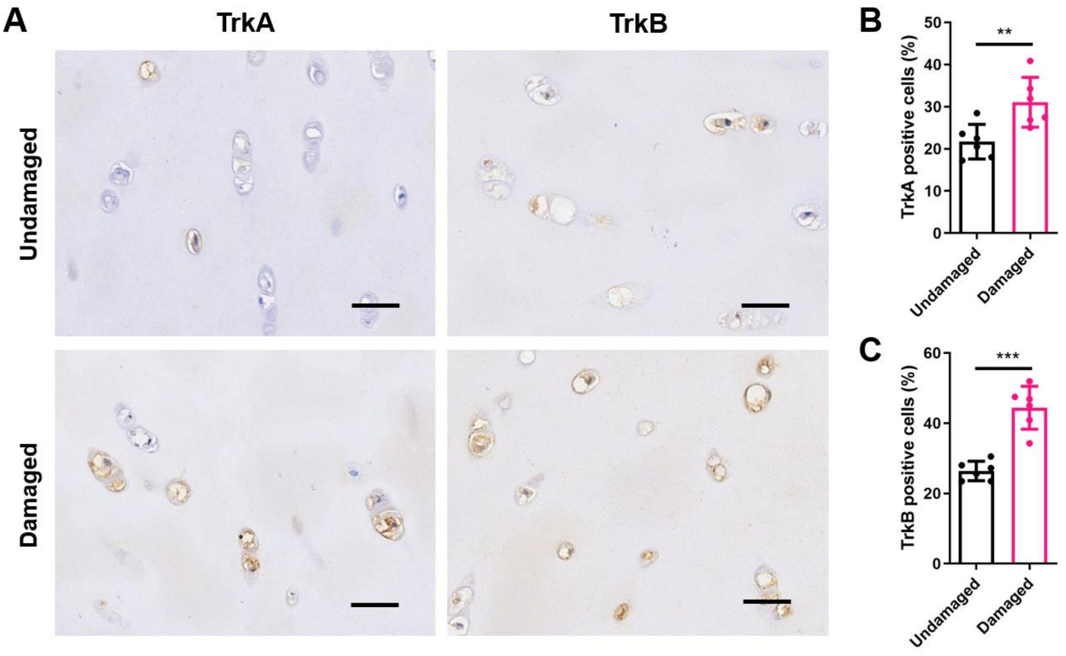

Fig. S4. The expression of TrkA and TrkB were upregulated in the damaged articular cartilage. (A) TrkA and TrkB immunohistochemical staining of undamaged and damaged articular cartilage samples from patients with OA (scale bar: 50 μm). (B, C) The proportion of TrkA and TrkB-positive cells in articular cartilage. $*P < 0.05$, $**P < 0.01$, $***P < 0.001$.

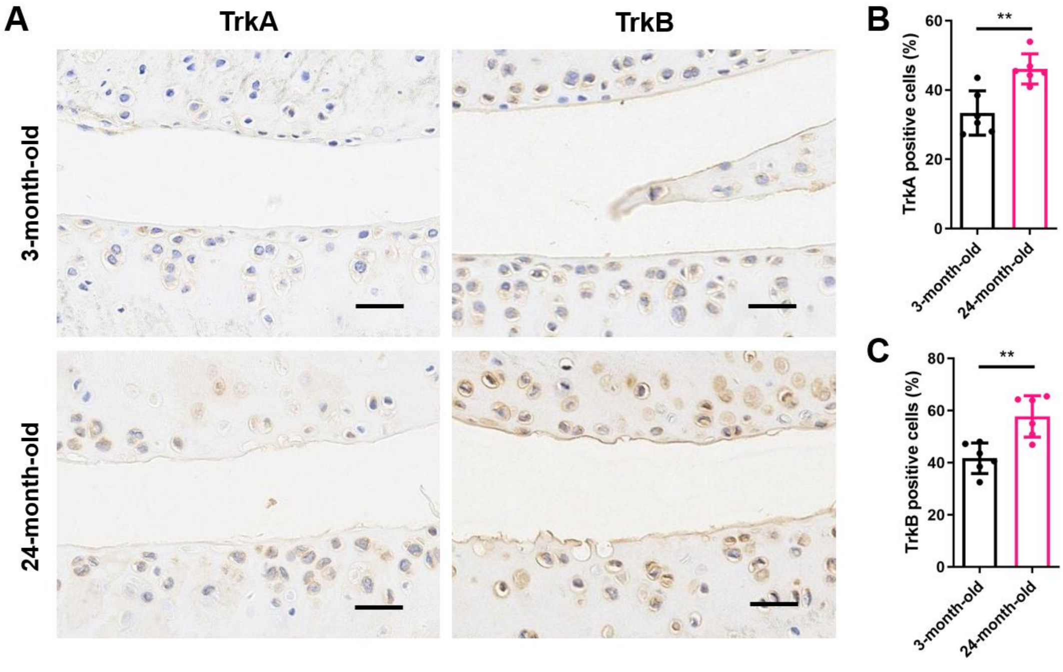

Fig. S5. The expression of TrkA and TrkB were increased in the articular cartilage of aging mice. (A) TrkA and TrkB immunohistochemical staining of articular cartilage of young and aging mice (scale bar: 20 μm). (B, C) The proportion of TrkA and TrkB-positive cells in articular cartilage. *P < 0.05, **P < 0.01, ***P < 0.001.

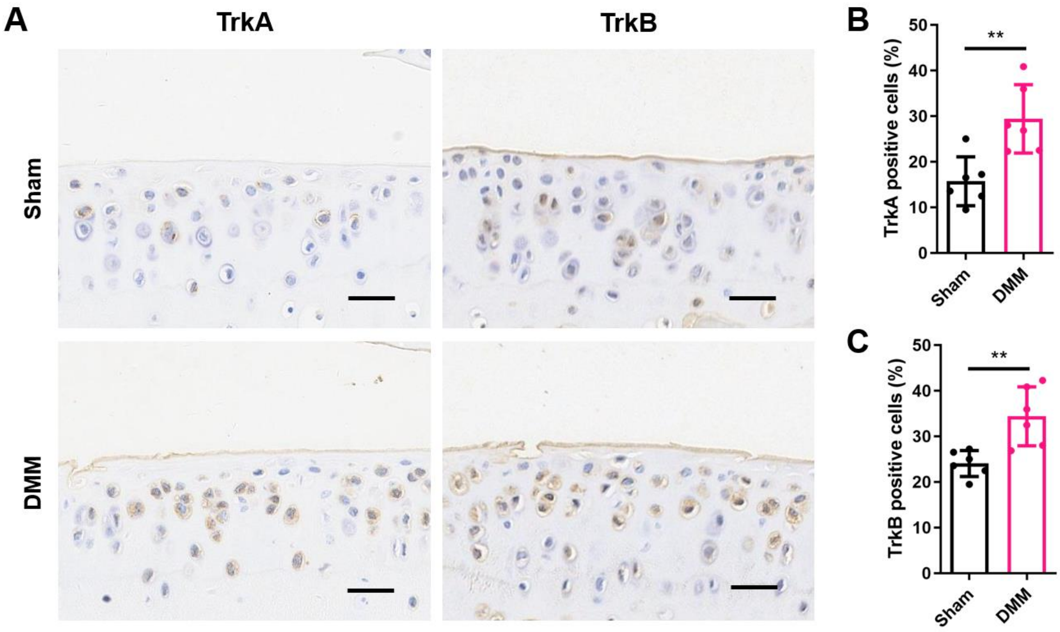

Fig. S6. The expression of TrkA and TrkB were elevated in the articular cartilage of DMM group. (A) TrkA and TrkB immunohistochemical staining of articular cartilage in sham and DMM groups (scale bar: 20 μm). (B, C) The proportion of TrkA and TrkB-positive cells in articular cartilage. *P < 0.05, **P < 0.01, ***P < 0.001.

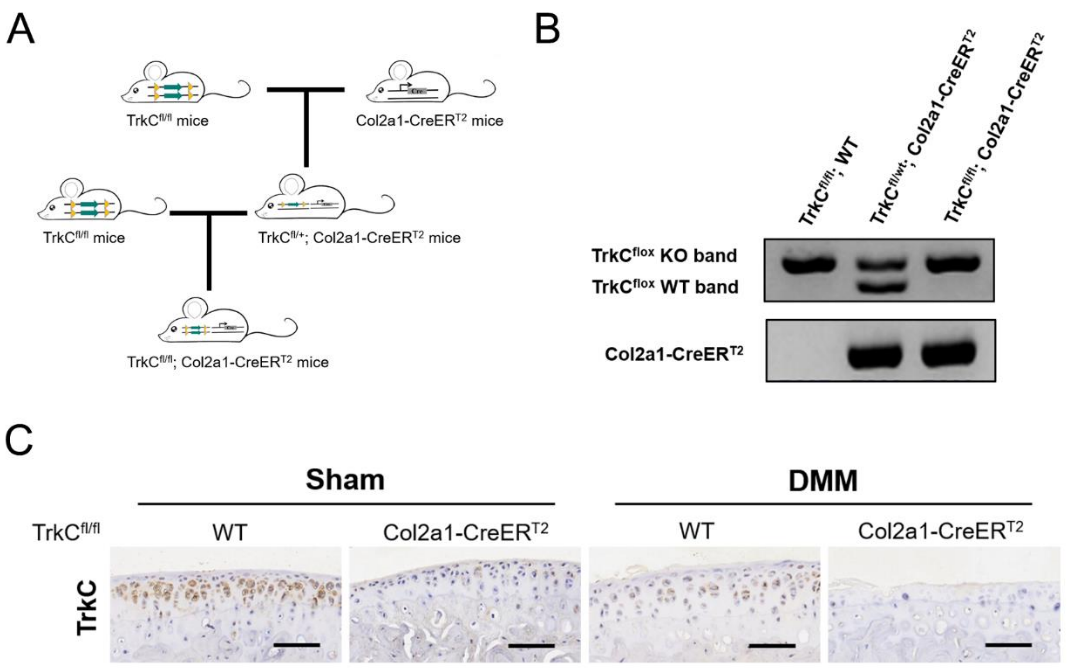

Fig. S7. The strategy, genotype identification and knockout efficiency of TrkC cKO mice. (A) The construction strategy of TrkC cKO mice. (B) The genotyping results of TrkC cKO mice. (C) The knockout efficiency of TrkC cKO mice was verified through immunohistochemical staining (Scale bar: 80μm).

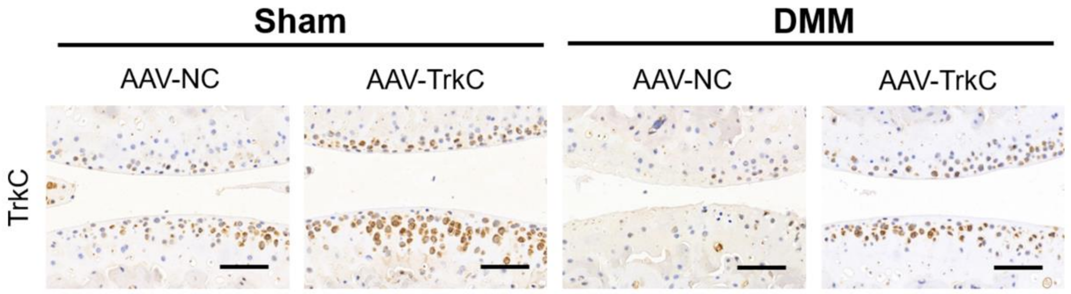

Fig. S8. The elevated expression of TrkC was verified through immunohistochemical staining in control and TrkC overexpression groups (Scale bar: 80μm).

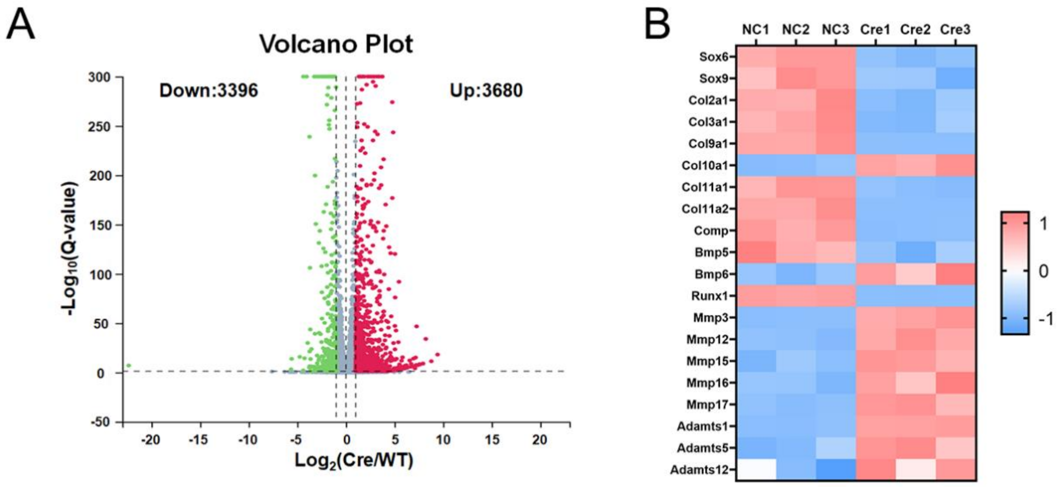

Fig. S9. The volcano plot and heatmap of differential gene expression between control and TrkC knockout chondrocytes. (A) The volcano plot of differentially expressed genes. (B) The heatmap of differentially expressed genes.

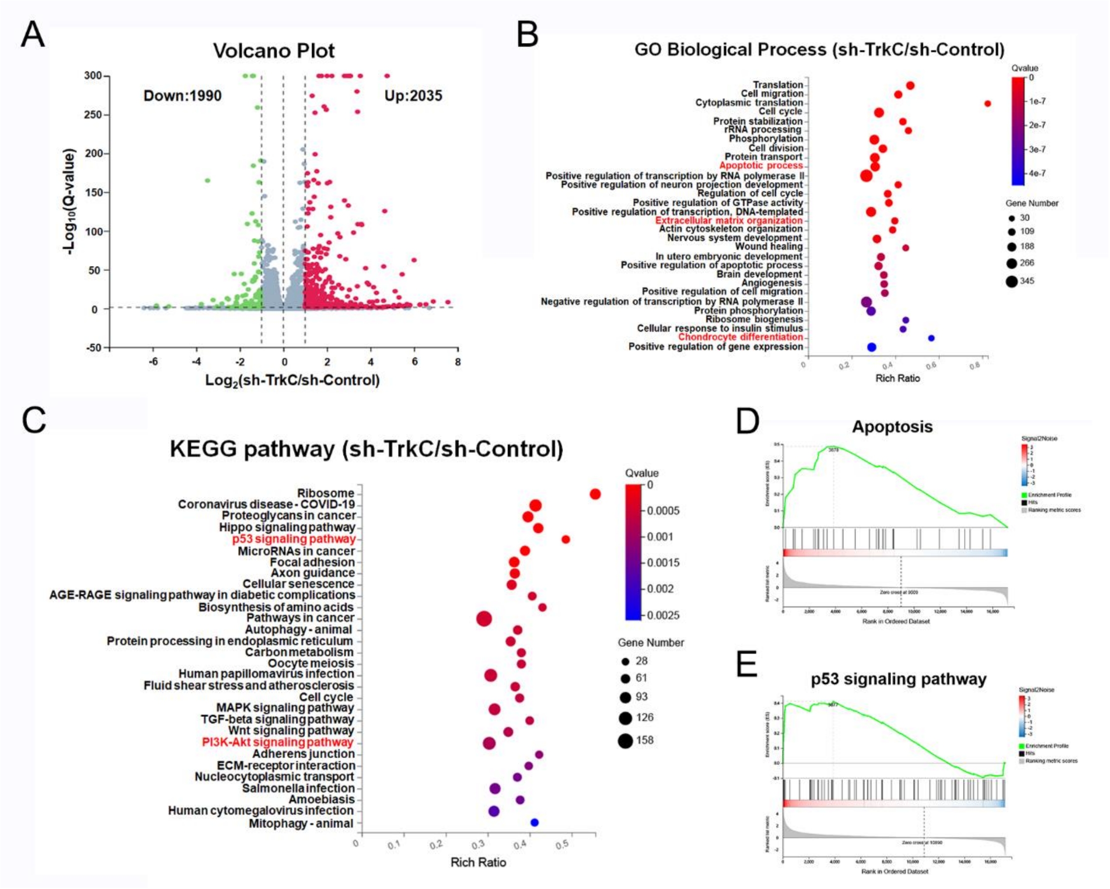

Fig. S10. The results of RNA sequencing between sh-NC and sh-TrkC groups. (A) Volcano plot of differentially expressed genes. (B) GO enrichment analysis. (C) KEGG enrichment analysis. (D, E) GSEA enrichment analysis.

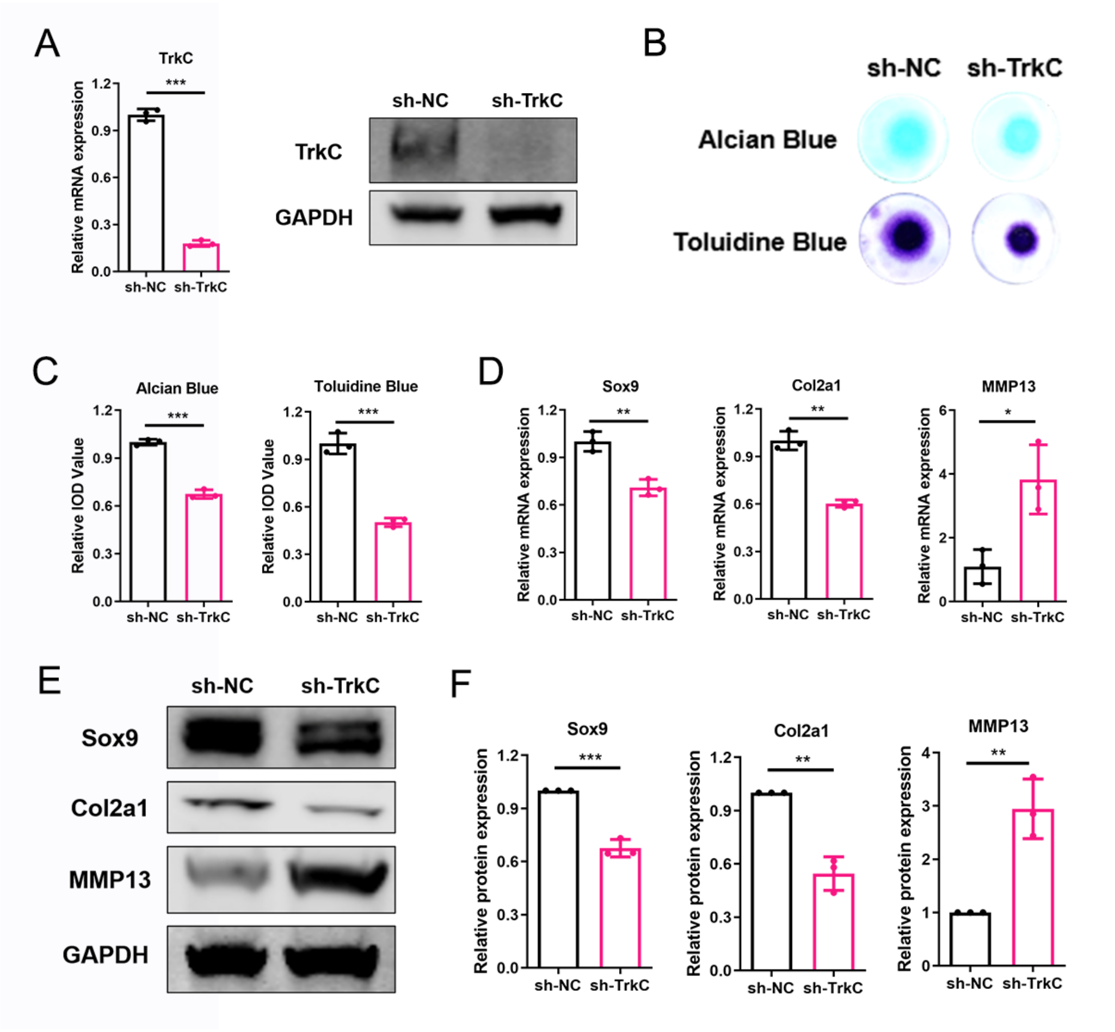

Fig. S11. TrkC silencing disturbed chondrocytes extracellular matrix metabolism. (A) Silencing efficiency of TrkC verified through PCR and WB. (B, C) Alcian blue and toluidine blue staining. (D) mRNA expression of chondrocytes extracellular matrix metabolism marker genes after silencing TrkC. (E, F) Expression of chondrocytes extracellular matrix metabolism related proteins after silencing TrkC. *$P < 0.05$, **$P < 0.01$, ***$P < 0.001$.

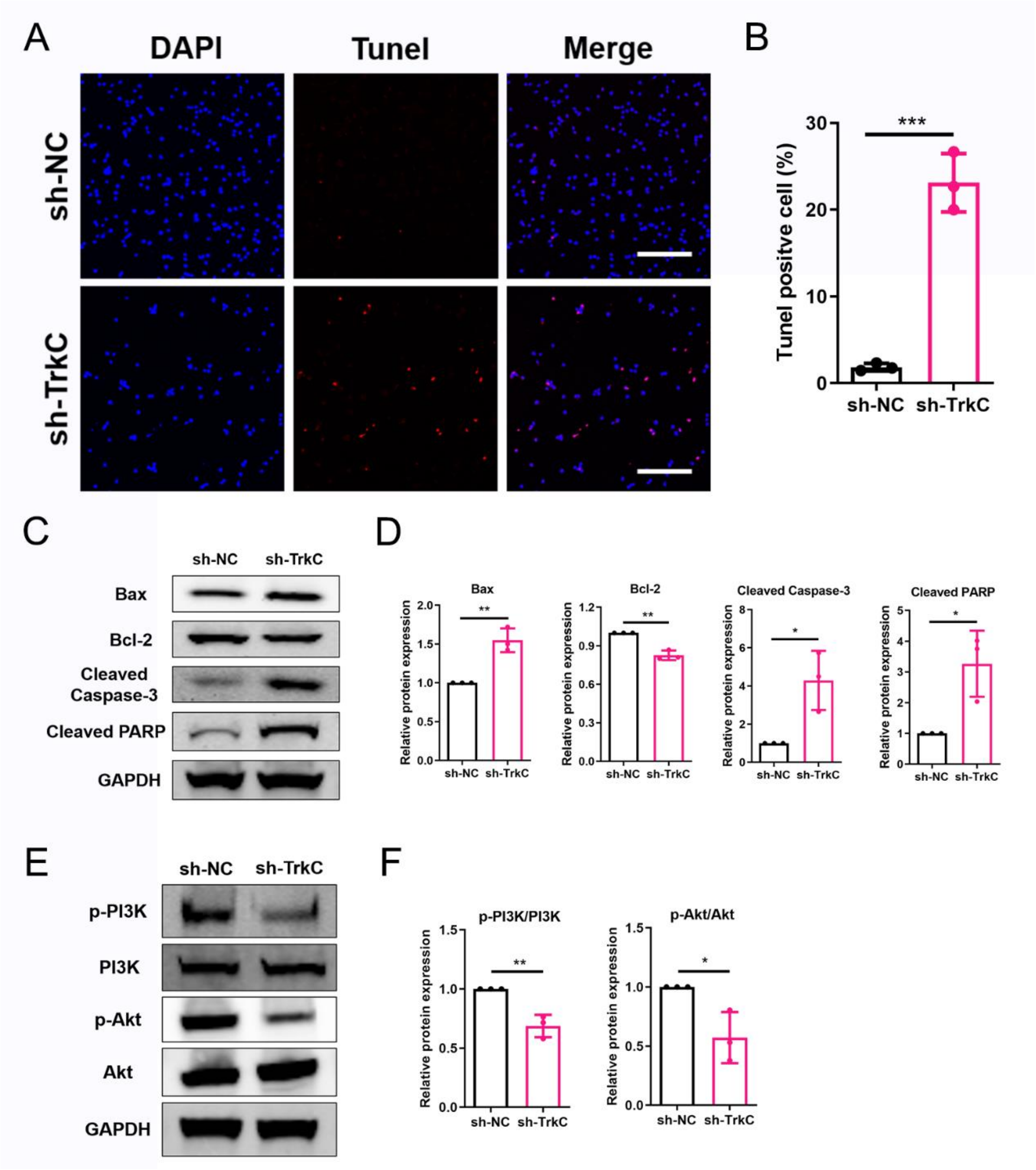

Fig. S12. TrkC silencing promoted chondrocytes apoptosis through PI3K/Akt signalling pathway. (A, B) Tunel fluorescence staining between sh-NC and sh-TrkC groups. Scale bar: 100μm. (C, D) Expression of chondrocytes apoptosis related proteins after silencing TrkC. (E, F) TrkC silencing decreased the protein expression of p-PI3K, and p-Akt (Ser473). *$P < 0.05$, **$P < 0.01$, ***$P < 0.001$.

Table S1 Primers used in the qRT-PCR assay

Genes	Forward (5′-3′)	Reverse (5′-3′)
TrkA	TGTCCAAGTCAGCGTCTCCT	AGCACAGAGCCGTTGAACAA
TrkB	GCCTACACGACGAACCTCTTG	GAGACAATGCCAGAAGCGAGTT
TrkC	GTGGCTGTTATCAGTGGAGAGG	ATGTGTCTGGCTTGTGGCAAT
Sox9	CGTGGACATCGGTGAACTGAG	GGTGCTGCTGATGCCGTAAC
Col2a1	GCTACACTCAAGTCACTGAACAACCA	TCAATCCAGTAGTCTCCGCTCTTCC
MMP13	GGAGCCCTGATGTTTCCCAT	GTCTTCATCGCCTGGACCATA
GAPDH	AGGTCGGTGTGAACGGATTTG	TGTAGACCATGTAGTTGAGGTCA

Table S2 The detailed information of antibodies

Antibodies	Source	Identifier
TrkC	Proteintech	11999-1-AP
Sox9	Abcam	ab185966
Clo2a1	Abcam	ab34712
MMP13	Abcam	ab39012
Bax	Cell Signaling Technology	#2772
Bcl2	Cell Signaling Technology	#3498
Cleaved-Caspase3	Cell Signaling Technology	#9661
Cleaved-PARP	Cell Signaling Technology	#9541
p-PI3K	Cell Signaling Technology	#4228
PI3K	Cell Signaling Technology	#4292
p-Akt	Cell Signaling Technology	#4060
Akt	Cell Signaling Technology	#4691
GAPDH	Cell Signaling Technology	#5174
Anti-rabbit IgG (H+L)	Cell Signaling Technology	#5151
